# Structural characterization of highly glucosylated crocins and regulation of their biosynthesis during flower development in *Crocus*

**DOI:** 10.3389/fpls.2015.00971

**Published:** 2015-11-04

**Authors:** Oussama Ahrazem, Angela Rubio-Moraga, Maria L. Jimeno, Lourdes Gómez-Gómez

**Affiliations:** ^1^Departamento de Ciencia y Tecnología Agroforestal y Genética, Facultad de Farmacia, Instituto Botánico, Universidad de Castilla-La ManchaAlbacete, Spain; ^2^Fundación Parque Científico y Tecnológico de Castilla-La ManchaAlbacete, Spain; ^3^Centro Química Orgánica “Lora-Tamayo” – Consejo Superior de Investigaciones CientíficasMadrid, Spain

**Keywords:** apocarotenoids, carotenoids, carotenoid cleavage dioxygenases, glucosylation, stigmas, tepals

## Abstract

Crocin biosynthesis in *Crocus* has been proposed to proceed through a zeaxanthin cleavage pathway catalyzed by carotenoid cleavage dioxygenase 2 (CCD2), and followed by glucosylation reactions catalyzed by CsGT2 (UGT74AD1). In *Crocus ancyrensis* flowers, crocins with eight (crocin-1), seven (crocin-2), and six glucose (crocin-3) moieties accumulated both in stigma and tepals. We have characterized the structure of these highly glucosylated crocins and follow up their accumulation by high-resolution liquid chromatography coupled with diode array detector along the development of both tissues, and coupled to the isolation and analysis of the expression of eighteen genes (*PSY-I*, *PSY-II*, *PDS-(I-V)*, *ISO-ZDS*, *ZDS*, *CtrISO*, *LYC-I* and *II*, *BCH*, *CaCCD2*, *UGT74AD2*-5) related with the apocarotenoid metabolism in *C. ancyrensis* tepals and stigmas. Structure elucidation of crocin-1 and crocin-2 was done by the combined use of 1D and 2D [^1^H, ^1^H] (gCOSY and TOCSY and ROESY) and [^1^H-^13^C] NMR experiments, revealing that for crocin-1 was all-*trans*-crocetin O-[β-D- Glucopyranosyl)-(1→4)-(β-D-glucopyranosyl)-(1→2)]-O-[β-D-glucopyranosyl-(1→6)]-β-D-glucopyranosyl diester, while crocin-2 showed an identical structure except for the absence of one glucose residue in one end of the molecule. Crocins accumulation was not synchronically regulated in stigma and tepals, although in both cases crocins accumulation parallels tissue development, decreasing at anthesis. The expression of the carotenogenic genes *PSY*, *ZDS-V*, *BCH*, and *LCY-II* was correlated with crocins accumulation. In addition, *CaCCD2* and only one of the four glucosyltransferase encoding genes, *UGT74AD2*, were highly expressed, and the expression was correlated with high levels of crocins accumulation in stigma and tepals.

## Introduction

Apocarotenoids are derived from the oxidative cleavage of carotenoids and constitute a growing class of secondary metabolites with important functions in animals, insects, microorganism, and plants (Britton, 2008). In higher plants, apocarotenoids act as phytohormones signaling molecules and provide color to flowers and fruits (Auldridge et al., 2006; Walter et al., 2010). Among these compounds, there are two economically important colored apocarotenoids produced by plants, crocetin, and bixin, used in the agro-food and in the pharmaceutical industry. Besides its color potential, crocetin has interesting biological properties that have been intensively studied with respect to its capacity in alleviating different diseases in humans (Hosseinzadeh and Nassiri-Asl, 2013). These health-promoting properties, along with its ability to act as natural colorant, have pushed an intense biotechnological interest to determine the biosynthetic pathways of crocetin in order to develop new, renewable sources producing these apocarotenoids (Winterhalter and Straubinger, 2000; Ahrazem et al., 2015).

**FIGURE 1:**
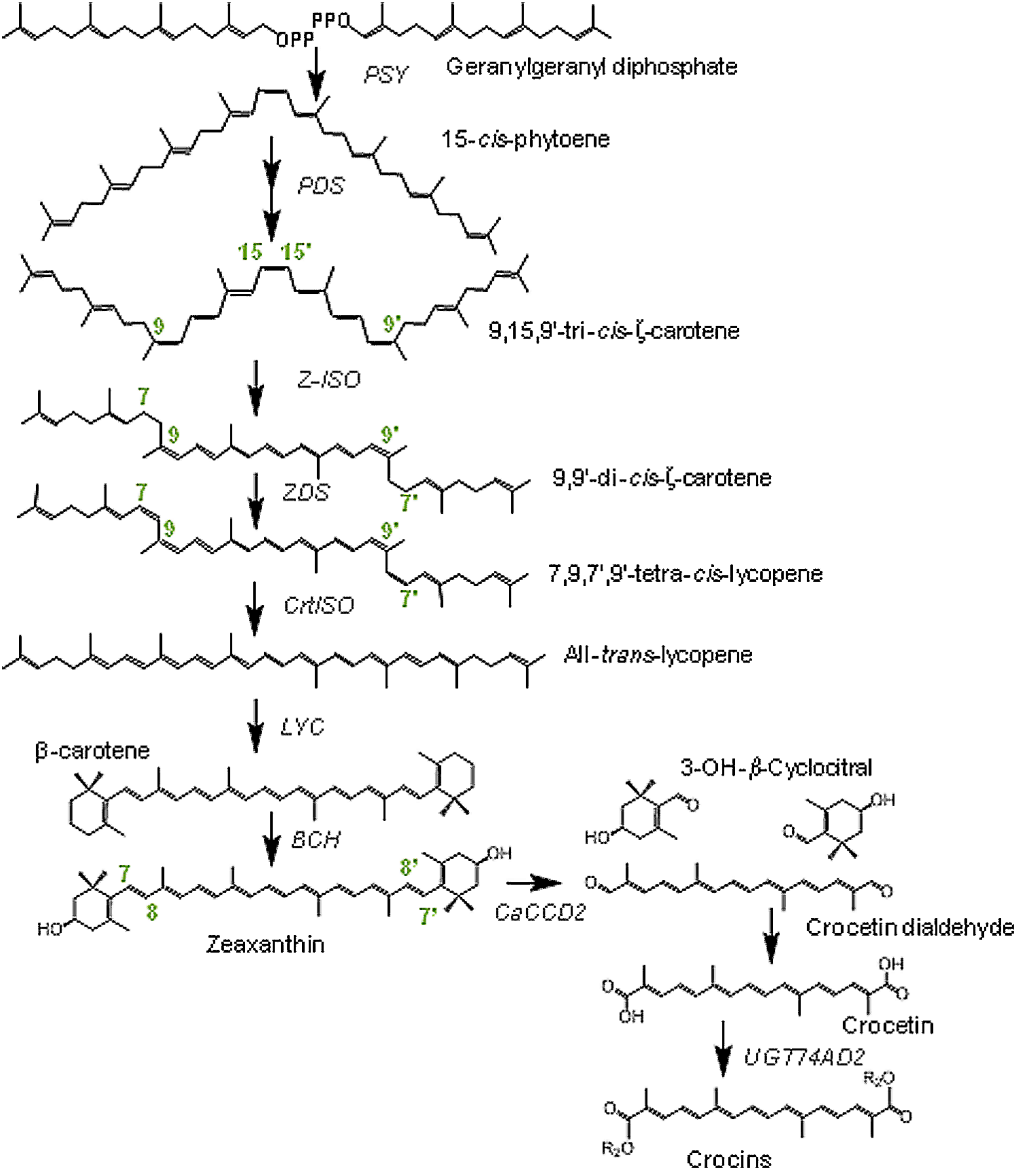
**Biosynthetic pathway for crocins in *Crocus ancyrensis* stigmas and tepals.** PDS, phytoene desaturase; PSY, phytoene synthase; Z-ISO,15-*cis*-ζ-carotene isomerase; ZDS, ζ-carotene desaturase; CtrISO, carotene isomerase; LCY, lycopene β-cyclase; BCH, β-carotene hydroxylase enzymes; CaCCD2, carotenoid cleavage dioxygenase 2 from *C. ancyrensis*.

The transcriptional regulation of carotenoid and crocetin pathways has been well documented in saffron stigmas (Castillo et al., 2005; Rubio et al., 2008; Moraga et al., 2009; Ahrazem et al., 2010). Biosynthetically, crocetin derives from zeaxanthin (**Figure 1**), synthesized in the chromoplast, using isopentenyl diphosphate (C5) derived from the methylerythritol-4-phosphate pathway (Pfander and Schurtenberger, 1982; Moraga et al., 2009). The conversion of two geranylgeranyl diphosphate (C20) molecules into phytoene (C40) represents the first committed step in the carotenoid pathway and is catalyzed by the enzyme phytoene synthase (**Figure 1**), (Cunningham and Gantt, 1998). Phytoene undergo a sequential series of desaturations and isomerizations to form all-*trans* lycopene. In plants, the desaturation and isomerization of phytoene to lycopene requires four proteins, a phytoene desaturase synthase and ζ-carotene desaturase, and two isomerases acting on ζ-carotene and poly *cis*-lycopene (Isaacson et al., 2002; Sandmann, 2009). Depending on the action and specificity of the cyclase enzymes, lycopene can then undergo cyclization to form β- or 𝜀-ionone rings, yielding β-carotene and/or α-carotene. These cyclic carotenoids can be further modified by hydroxylation and epoxidation reactions (Nisar et al., 2015). β-carotene hydroxylation in both rings generates zeaxanthin, the precursor of crocetin (**Figure 1**). Zeaxanthin cleavage at the 7,8;7′,8′ double bonds by the carotenoid cleavage dioxygenase 2 (CCD2; Frusciante et al., 2014) generates crocetindial and two molecules of 3-hydroxy-β-cyclocitral. Both apocarotenoids are substrate of glucosyltransferases that transform them in crocins and picrocrocin, respectively (Moraga et al., 2004; Nagatoshi et al., 2012). Several crocins have been identified in saffron as crocetin mono (β-D-glucosyl) ester, crocetin di (β-D-glucosyl) ester, crocetin mono (β-gentiobiosyl) ester and crocetin (β-D-glucosyl) (β-gentiobiosyl) ester and crocetin (β-gentiobiosyl) (β-neapolitanosyl) ester (Pfister et al., 1996; Moraga et al., 2009).

The main commercial sources of crocins are the stigma of *Crocus sativus* and the fruit of *Gardenia Jasminoides*. However, the ability to synthesized crocetin is not restricted to these species (Moraga et al., 2009; Rubio Moraga et al., 2013). In particular, crocetin is widely present inside the genus *Crocus* (Castillo et al., 2005), and in certain spring-flowering species crocetin is not restricted to the stigma but also present in tepals (Rubio Moraga et al., 2013). Interestingly, these species are characterized by the presence of crocins containing more glucose molecules than those from other autumn *Crocuses* (Castillo et al., 2005; Rubio Moraga et al., 2013). Crocins with up to eight glucose molecules have been detected although its arrangement and configuration remains unresolved.

To make further progress on the structure, formation, and accumulation of these highly glucosylated crocins in spring *Crocuses* we purified these crocins and resolved their structure. Further, we followed the pattern of synthesis and accumulation of crocins and the expression of carotenoid- and apocarotenoid-related genes during stigma and tepal development in order to determine the factors influencing accumulation of these bioactive molecules and to identify key steps in their biosynthetic pathway.

## Materials and Methods

### Chemicals and Plant Materials

Chemicals and reagents were obtained from Sigma–Aldrich unless otherwise stated. Tepals and stigma were obtained from *C. ancyrensis* grown under field conditions in the Botanical Garden of CLM (Albacete, Spain). The tissues were collected at different developmental stages frozen in liquid nitrogen and stored at –80°C until required.

### Nucleic Acid Purification and cDNA Isolation

Total RNA was isolated from *C. ancyrensis* stigma and tepals at eight developmental stages by using RNeasy Plant Mini Kit, following manufacturer’s protocols (Qiagen, Hilden, Germany). First-strand cDNAs were synthesized by reverse transcription (RT) from 2 μg of total RNA using an 18-base pair oligo dT primer and a first-strand cDNA synthesis kit (GE Healthcare Life Sciences, Buckinghamshire, UK) according to manufacturer’s instructions. These cDNAs were used as templates for PCR using primers designed from genes related to carotenoid metabolism from *C. sativus* (Supplementary Table S1). Conditions for RT were as follows: 65°C for 5 min, followed by 37°C for 1 h, followed by 75°C for 5 min. Thermal cycling parameters were 2 min at 95°C, 35x (30 s at 95°C, 20 s at 60°C and 1min at 72°C) and finally 5 min at 72°C. The full-length clones were obtained by a reverse transcription-polymerase chain reaction of 3′ and 5′ amplification ends (SMARTer^TM^ RACE cDNA Amplification Kit, Clontech, Palo Alto, CA, USA) using RNA from stigma tissue, and several primer combinations (Supplementary Table S1). The PCR products were separated in 0.8% agarose gel stained with ethidium bromide, purified, ligated into pGEMT-easy (Promega, Madison, WI, USA) and then introduced into *Escherichia coli*.

### DNA Sequencing and Analysis of DNA and Protein Sequences

Plasmids were sequenced with M13 reverse and forward primers using an automated DNA sequencer (ABI PRISM 3730xl, Perkin Elmer, Macrogen Inc., Seoul, Korea). Sequence similarity searches were made with the BLAST suite of programs of the National Centre for Biotechnology Information (NCBI^1^). Protein sequences were analyzed using SignalP 4.1^2^ and PSORT II^3^.

### Phylogenetic Analysis

Amino acid sequences were used to construct the phylogenetic trees. The sequences were aligned using the BLOSUM62 matrix with the ClustalW^4^ algorithm-based AlignX module from MEGA Version 6.0^5^. The obtained alignments were saved and executed by MEGA Version 6.0 to generate a Neighbor Joining Tree with bootstrapping (5000 replicates) analysis and handling gaps with pairwise deletion.

### Gene Expression by Quantitative Reverse Transcription-PCR (qRT-PCR)

Gene-specific oligonucleotides were used for the expression analysis (Supplementary Table S1), using the first-strand cDNAs obtained from the developmental stages selected from tepals and stigma. The qRT-PCR was carried out on cDNA from three biological replicates; reactions were set up in a final volume of 25 μl in GoTaq^®^ qPCR Master Mix (Promega, Madison, WI, USA) according to manufacturer’s instructions. The constitutively expressed 18SrRNA gene was used as a reference gene (Rubio-Moraga et al., 2014). The cycling parameters consisted in an initial denaturation at 94°C for 5 min; 40 cycles of denaturation at 94°C for 20 s, annealing at 58°C for 20 s and extension at 72°C for 20 s; and a final extension at 72°C for 5 min. Assays were conducted with a StepOne^TM^ Thermal Cycler (Applied Biosystems, Foster City, CA, USA) and analyzed using StepOne software v2.0 (Applied Biosystems, Foster City, CA, USA).

### Extraction and Analysis of Carotenoids and Apocarotenoids by HPLC-DAD

Frozen stigma and tepals at different developmental stages were ground in liquid nitrogen with the mixer mill MM400 (Retsch GmbH, Haan, Germany) in a 1.5 ml Eppendorf tube, and then extracted with 1 ml Tris-HCl (50 mM, pH 7.5; containing 1 M NaCl), and incubated for 10 min on ice. One volume of CHCl_3_ was then added, mixed, and the extract incubated on ice for an additional 10 min followed by centrifugation at 3,000 *g* for 5 min at 4°C. The lower CHCl_3_ phase was evaporated under N_2_ gas and the dried residues were stored together with the upper aqueous phases at –80°C until analysis by HPLC. All assays were performed in triplicate.

The HPLC methods used for the analysis and detection of glycosylated apocarotenoids and carotenoids have been previously described (Castillo et al., 2005; Rubio Moraga et al., 2013).

### NMR Structure Characterization

Structure elucidation was accomplished by nuclear magnetic resonance spectroscopy (NMR). NMR spectra were recorded at 298 K, using D_2_O as the solvent, on a Varian SYSTEM 500 NMR spectrometer (^1^H 500 MHz, ^13^C 125 MHz) equipped with a 5 mm HCN cold probe. Chemical shifts of ^1^H (δH) and ^13^C (δC) in ppm were determined relative to an external standard of sodium [2, 2, 3, 3-2H4]-3-(trimethylsilyl)-propanoate in D_2_O (δH 0.00 ppm) and 1,4-dioxane (δC 67.40 ppm) in D_2_O, respectively. One-dimensional NMR experiments (^1^H,) were performed using standard Varian pulse sequences. Two-dimensional [^1^H, ^1^H] NMR experiments (gCOSY, TOCSY, and ROESY) were carried out with the following parameters: a delay time of 1 s, a spectral width of 4310.3 Hz in both dimensions, 4096 complex points in t2 and 8 (gCOSY, TOCSY) or 64 (ROESY) transients for each of 200 time increments, and linear prediction to 512. The data were zero-filled to 4096 × 4096 real points. Two-dimensional [^1^H-^13^C] NMR experiments (gHSQC and gHMBC) used the same ^1^H spectral window, a ^13^C spectral windows of 25133 Hz, 1 s of relaxation delay, 1024 data points, and 128 time increments, with a linear prediction to 256. The data were zero-filled to 4096 × 4096 real points. Typical numbers of transients per increment were 32 and 64, respectively.

## Results

### Structural Characterization of Highly Glucosylated Crocins in *C. ancyrensis* Tepals and Stigma

Previous analytical data of tepals and stigma aqueous extracts indicated the presence of several crocin compounds with crocetin as the aglycone and between eight and two units of glucose (Rubio Moraga et al., 2013). The main component, crocin-1 (Supplementary Figure S1) was isolated, and the unequivocal structural elucidation of this compound was carried out by the combined use of 1D and 2D [^1^H, ^1^H] (gCOSY and TOCSY and ROESY) and [^1^H-^13^C] NMR experiments (multiplicity-edited gHSQC and gHMBC). The ^1^H and ^13^C NMR data (Supplementary Table S2) indicated that the structure of crocin-1 was in accordance with a crocetin ester with 8 units of glucose. The ^1^H NMR spectrum revealed two methyl singlets at δ 1.88 and 1.87, an ABC system at δ 7.35 (d, *J* = 11.3 Hz, H3), 6.59 (dd, *J* = 15.0, 11.3 Hz, H4) and 6.71 (d, *J* = 15.0 Hz, H5), and an AB system at δ 6.76 (d, *J* = 10.0 Hz, H8), 6.41 (d, J = 10.0 Hz, H7), corresponding to the aglycone moiety (Pfister et al., 1996; Choi et al., 2001). In addition, four anomeric proton signals at δ 5.66 (d, *J* = 7.8 Hz), 4.57 (d, *J* = 8.1 Hz), 4.32 (d, *J* = 7.9 Hz), and 4.30 (d, *J* = 7.9 Hz) were observed. The ^1^H NMR spectrum showed a number of signals indicated the existence of a twofold axis of symmetry. Hence, R1 and R2 substituents must be equal, corresponding to tetrasaccharide moieties.

The TOCSY spectrum showed in Supplementary Figure S2A allowed the assignment of the sequential order of the chemical shifts belonging to the same spin system. In conjunction with the gHSQC and gHMBC spectra the full assignment of ^1^H and ^13^C of crocin-1 was carried out.

The TOCSY H1-A trace at δ 5.66 (*J* = 7.8 Hz) showed the scalar coupling network H2(t),H3(t), H4(t), H5(m), H-6a(d), and H-6b(d). The^13^C chemical shifts were measured from the gHSQC spectrum. The chemical shifts of H1-A and C1-A indicated that this moiety is bonded to the aglycone (Dufresne et al., 1999; Hong and Yang, 2013). The downfield shifts observed in the ^1^H spectrum for H2-A, H6a-A, and H6b-A combined with the same effect in the ^13^C spectrum (C-2 δ80.9; C-6 δ67.8), and the gHMBC correlations between H1-B and C2-A, and between H1-D and C6-A confirmed that residue A corresponds to a (2,6)-β-D-Glcp- unit, linked to the ester of the crocetin moiety. NMR chemical shifts for this fragment are in agreement with those published by (Wang et al., 1999).

The TOCSY H1-B trace at δ 4.57 (*J* = 8.1Hz) showed the scalar coupling network H2 (t),H3-H4(m), H5(m), H6a(d), and H-6b(d). The downfield shift observed in the ^13^C spectrum for C4 and the gHMBC cross-peaks between H1-B and C2-A, and between H4-B and C1-C suggested that this fragment has an extra substitution in C4. Therefore, residue B corresponds to a (1,4)- β-D-Glcp unit linked to fragment A.

The TOCSY H1-C trace at δ 4.30 (*J* = 7.9Hz) and H1-D trace at δ 4.32 (*J* = 7.9Hz) showed very similar scalar coupling networks [H2(t),H3(t), H4(t), H5(m), H6a(d), and H-6b(d)], and their values were in agreement with those published by Roslund et al. (2008) for β-D-(1-4)-and β-D-(1-6)-β-D-Glcp. The gHMBC correlations observed between H1-C and C4-B and between H1-D and C6-A confirmed the tetrasaccharide structure.

**FIGURE 2:**
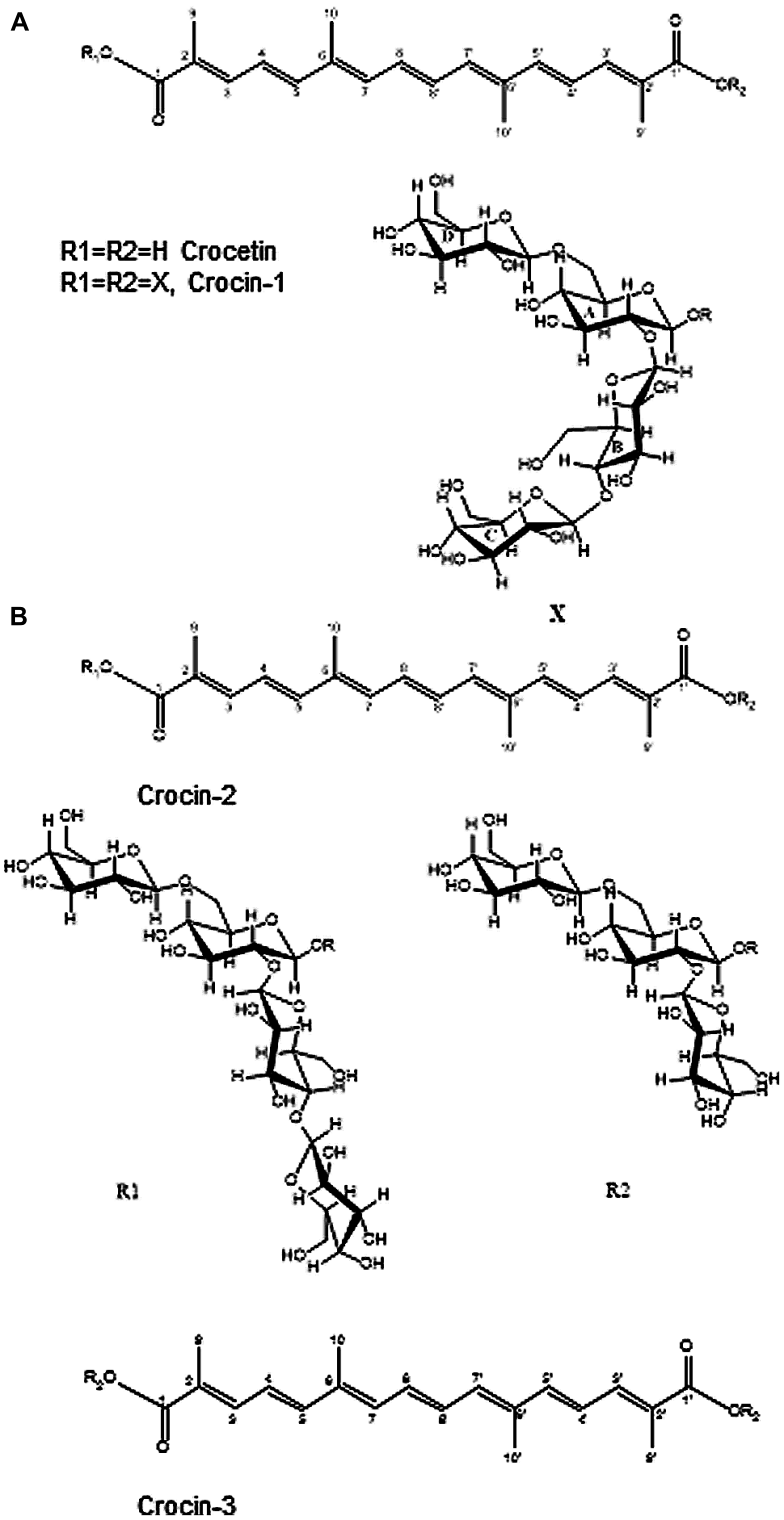
**Structure of highly glucosylated crocins in *Crocus ancyrensis* flowers. (A)** Structure of crocin-1. **(B)** Structures of crocin-2 and crocin-3.

Finally, a ROESY experiment was carried out in order to confirm the new structure as all-*trans*-crocetin *O*-[β-D-Glucopyranosyl)-(1→4)-(β-D-glucopyranosyl)-(1→2)]-*O*-[β-D-glucopyranosyl-(1→6)]-β-D-glucopyranosyl diester (**Figure 2A**). In the Supplementary Figure S2B the relevant correlations obtained are shown.

In addition, a minor component crocin-2 was also isolated and analyzed by NMR. Due to the small sample quantity, only 1D- and 2D-^1^H NMR experiments were performed. The ^1^H NMR spectrum of crocin-2 showed the same signals as crocin-1 for the aglycone. Besides the presence of seven glucose anomeric protons [two of them at δ 5.67 (d, *J* = 7.8 Hz), and at δ 5.66 (d, *J* = 7.8 Hz) involved in two ester linkages with the crocetin moiety], suggested the non- existence of symmetry in the molecule. From comparison of 1H-NMR data (1D- TOCSY and ROESY spectra), with those for the crocin-1, we determined the crocin-2 structure almost identical to crocin-1 except for the absence of one glucose residue in one end of the molecule (**Figure 2B**).

### Crocins Evolution during Stigma and Tepals Development

**FIGURE 3:**
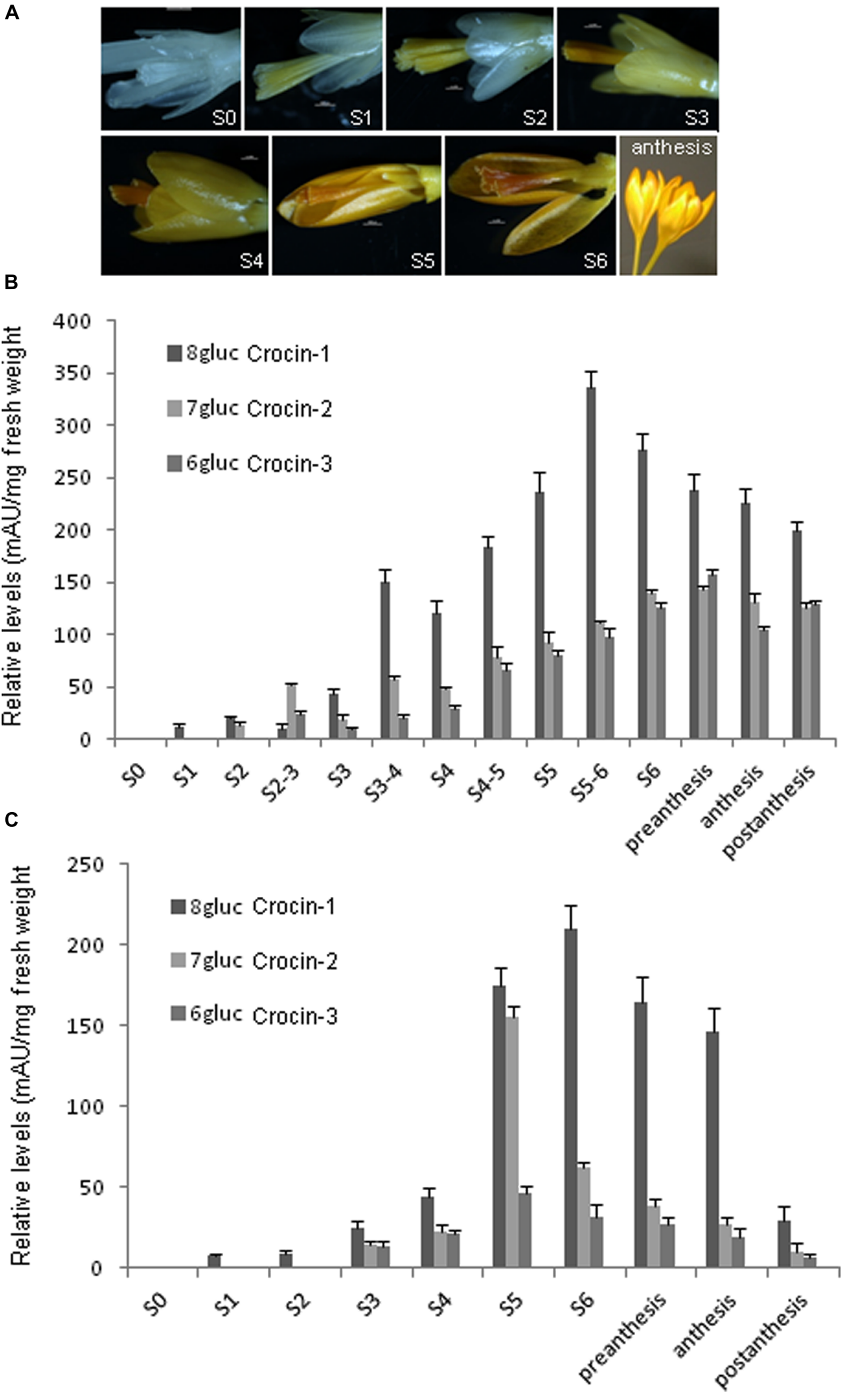
**Stigma and tepal development is characterized by the increasing yellow to orange coloration, due to crocins accumulation, until the flower reaches the anthesis stage. (A)** Eight developmental flower stages characterized by color development. Stage 0 (S0): white stigma and white tepals; stage 1 (S1): yellowish stigma and white tepals; stage 2 (S2): yellow stigma and yellowish tepals; stage 3 (S3): orange stigma and yellow tepals; stage 4 (S4): dark orange stigma and dark yellow tepals; stage 5 (S5): dark orange stigma and orange tepals; stage 6 (S6): dark orange stigma and dark orange tepals; anthesis, open flower. Scale bars S1–S6: 1000 μm. **(B)** Levels of crocins with eight (crocin-1), seven (crocin-2), six (crocins-3), and two glucose molecules in stigma at different developmental stages. **(C)** Levels of crocins with eight (crocin-1), seven (crocin-2), six (crocin-3), and two glucose molecules in tepals at different developmental stages.

Stigma and tepal development is characterized by the increasing yellow to orange coloration until the flower reaches the anthesis stage (**Figure 3A**). This process does not take place synchronically in both tissues. The stigma developed faster than the tepals and also the accumulation of crocins began earlier (**Figures 3A,B**). Fourteen and ten points were selected along the development of stigma and tepals, respectively, and analyzed for the content on *trans*-crocin with eight (crocin-1), seven (crocin-2), and six (crocin-3) glucose molecules (**Figures 2B** and **3B,C**). In stigma, from stage S1 onward there is a predominance of crocin-2 and crocin-1. The highest concentration of crocin-1 is reached at stage S4 (**Figure 3B**). While crocin-2 is the most abundant crocin in S2-3. The crocins profile is different in the case of tepals, where crocin-1 is predominant in all the developmental stages analyzed (**Figure 3C**).

### Isolation and Expression Analysis of Genes Involved in the Biosynthesis of Crocetin Precursors

In order to envisage the mechanism controlling the accumulation of zeaxanthin, the crocetin precursor, in tepals and stigma of *C. ancyrensis*, we cloned and analyzed the expression of 12 carotenogenic genes during the stigma and tepal development. Two genes with identity to phytoene synthase genes, *CaPSY-I* and *CaPSY-II* (Supplementary Figure S3 and Table S3); five genes with identities to putative phytoene desaturase genes (*CaPDS-I* to *CaPDS-V*; Supplementary Figure S3 and Table S3); one gene with identity to putative ζ-carotene desaturase genes, *CaZDS* (Supplementary Figure S3 and Table S3); one gene with identity to putative *CtrISO*, Ca*CtrISO* (Supplementary Figure S3 and Table S3) one gene with identity to putative 15-*cis*-ζ-carotene isomerase, *CaZISO* (Supplementary Figure S4 and Table S3); two genes with identity to putative lycopene β-cyclase, *CaLCYB-I* and *CaLCYB-II* (Supplementary Figure S4 and Table S3) and one gene with identity to β-carotene hydroxylase, *CaBCH* (Supplementary Figure S4 and Table S3); were identified in *C. ancyrensis*.

Phylogenetic analyses showed that the carotenogenic enzymes isolated are closely related to the enzymes from other monocotyledonous species (Supplementary Figures S3 and S4), with the exception of CaBCH. The BCH enzymes from *C. ancyrensis* and *C. sativus* cluster together in a separate group from the monocot and dicot sequences. Sequence alignment showed that the main differences observed are present at the N-terminal region of the proteins (Supplementary Figure S5).

The presence of putative hydrophobic domains in the structure of the isolated genes from *C. ancyrensis* was investigated using the TMHMM Server v. 2.0 (Supplementary Figure S6). Hydrophobic domains were detected in the protein sequences of CaPSY-I, CaPSY-II, CaPDS-II, CaPDS-IV, CaZISO, and CaBCH, while not obvious hydrophobic domains were predicted in the primary structure of the other enzymes (Supplementary Figure S6). Furthermore, in CaZISO and CaBCH several putative transmembrane domains were predicted (Supplementary Figure S6). Comparison of these hydrophobic domains with those from carotenogenic enzymes from other plants species, allowed stablishing different hydrophobic profiles for PSY isoforms (Supplementary Figure S7). The maize and rice PSY1 isoenzymes showed a similar profile to CaPSY-I and CaPSY-II, whereas Narcissus PSY1, maize PSY2 and PSY3 showed a different hydrophobic profile, with a predicted transmembrane domain.

**FIGURE 4:**
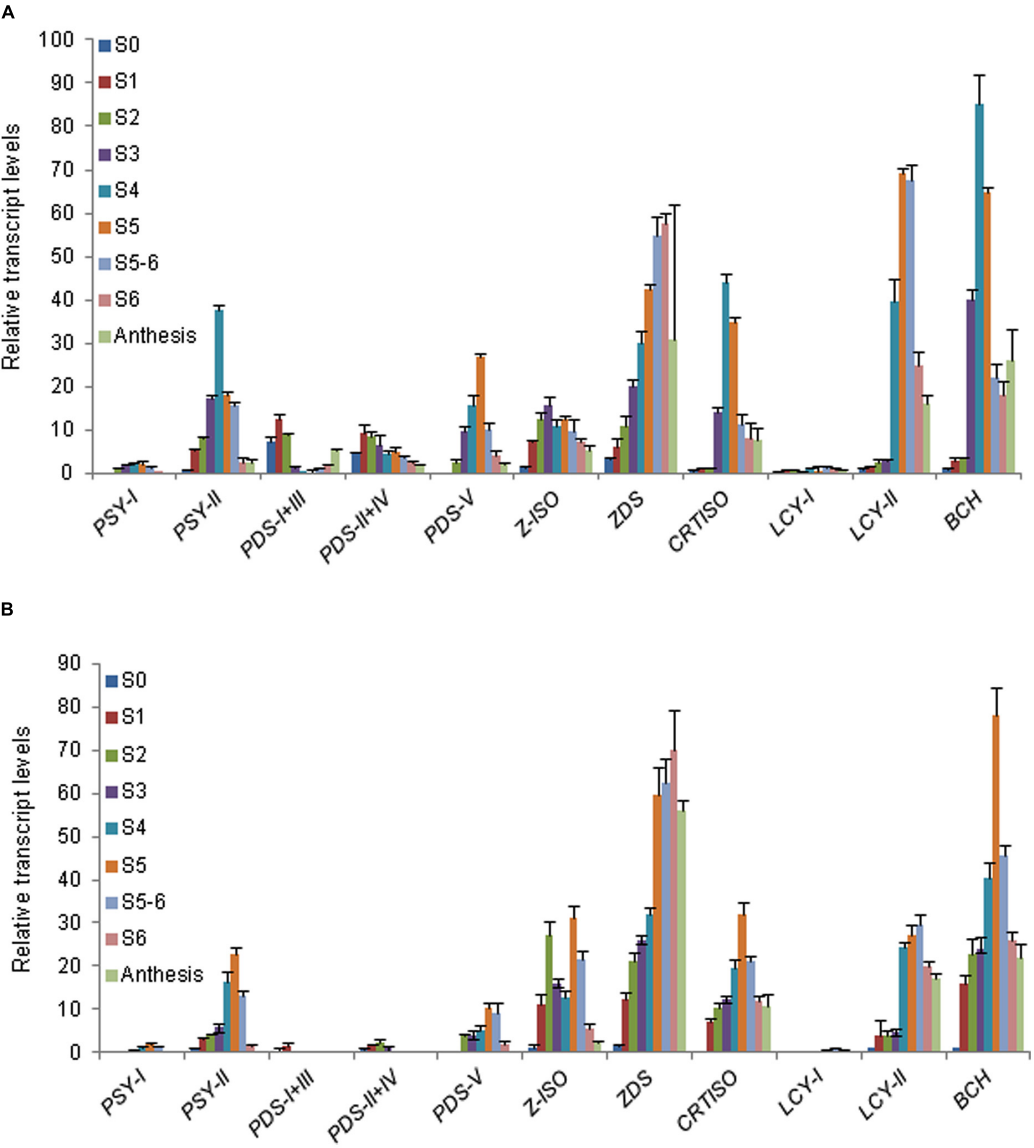
**Analysis of carotenogenic genes expression during stigma and tepal development in flowers of *C. ancyrensis*. (A)** Relative expression levels in nine developmental stages of stigma. **(B)** Relative expression levels in nine developmental stages of tepals. Values are means of three technical replicates ±SE, normalized to the internal control gene.

The expression of all these carotenogenic genes was tested in nine developmental stages of stigma and tepals (**Figure 4**). Due to the high sequence identity between CaPDS-I and III and between PDS II and IV (Supplementary Figure S8) it was not possible to design specific oligonucleotides to discriminate their expression patterns. Therefore, *CaPDS-I* is presented together with *CaPDS-III* and *CaPDS-II* with *CaPDS-IV*.

*CaPSY-I* was expressed at low levels in stigma and tepals. By contrast, *CaPSY-II* levels were up to 10-fold higher. The highest expression level of *CaPSY-II* in stigma was reached in the S4 stage while in tepals was reached in S5 (**Figures 4A,B**). In general, the expression levels of *CaPDS* were higher in stigma than in tepals. However, both in stigmas and tepals, *CaPDS-I/III* and *CaPDS-II/IV* showed the highest expression levels in the earlier developmental stages (**Figure 4** and Supplementary Figure S9), and their expression is down-regulated in those developmental stages characterized by the accumulation of apocarotenoids (**Figure 3**). Interestingly, *CaPDS-I/III* levels were reduced during the stages of higher crocins accumulation, and increased again in the late developmental stages, coincident with the decreased on crocins accumulation in the stigma (Supplementary Figure S9), a similar profile was observed in tepals, although at much reduced levels (Supplementary Figure S9). An opposite behavior is found for *CaPDS-V*, whore expression levels parallels apocarotenoids accumulation (**Figures 3** and **4**), suggesting the involvement of *CaPDS-V* in the biosynthesis of these compounds in stigma and tepals. The expression of *CaZISO* clearly differed between stigma and tepals (**Figure 4**). Whereas in stigma its expression levels increased from S0 to S4 and decreased thereafter, in tepals *CaZISO* showed two peaks of expression, at S2 and S5. In tepals, from S2 to S3 takes place a seven fold increase in the crocin content and in S5 the highest levels of crocins are detected (**Figure 3**). *CaZDS* expression levels showed a similar behavior in stigma and tepals, with a continuous increase from S0 to S6, and decreasing at anthesis (**Figure 4**). *CaCtrISO* showed an expression profile in stigma and tepals similar to *CaBCH*, with a maximum in stigma at S4 and in tepals at S5 (**Figure 4**). Finally the expression levels of the *CaLCY-I* and *CaLCY-II* were also evaluated; being the expression of the chromoplast-specific *CaLCY-II* much higher than the expression levels of *CaLCY-I*, with maximum levels of expression at S5 and S5-6 in stigma and tepals, respectively (**Figures 4A,B**).

### Expression Analysis of CaCCD2 Involved in Crocetin Biosynthesis

**FIGURE 5:**
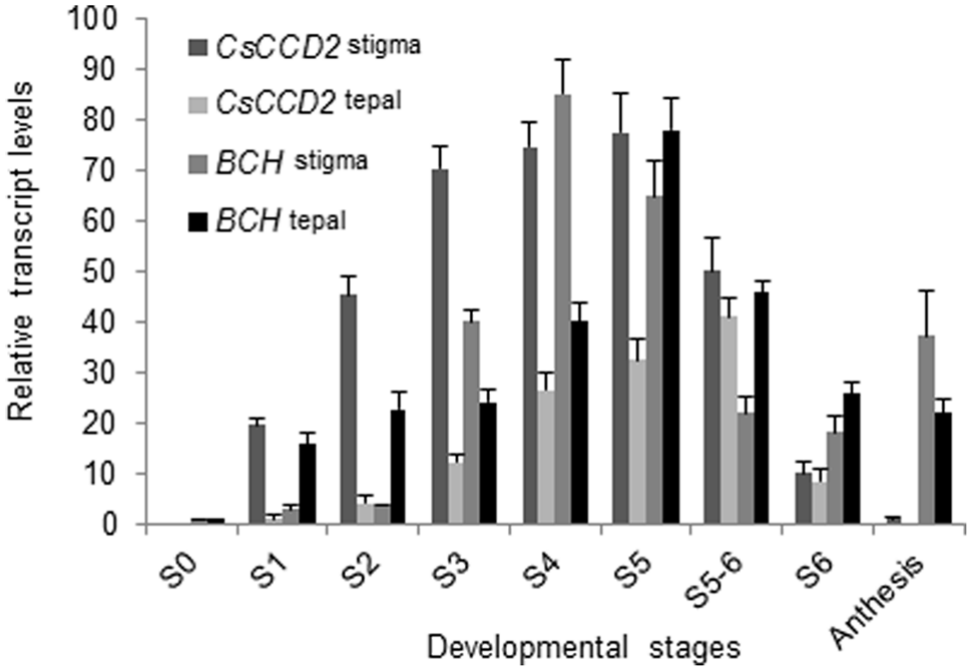
***CaCCD2* transcript accumulation (normalized to the household gene *RSP18*) in the flowers tissues of *C. ancyrensis* along their development.** Values are means of three technical replicates ±SE, normalized to the internal control gene. The relative expression levels of *BCH* were incorporated for comparison.

We determined the expression levels of *CaCCD2*, encoding for the enzyme responsible for zeaxanthin cleavage and crocetin formation in *C. ancyrensis*, in the stigma and tepals along their development. In stigma *CaCCD2* expression levels were increased from S1 to S5, and decreased after this developmental stage (**Figure 5**). In tepals *CaCCD2* levels were much reduced than in stigma, reaching the highest level in stages S5–S6 (**Figure 5**).

### Isolation and Analysis of *C. ancyrensis* Genes Implicated in Crocin Biosynthesis

**FIGURE 6:**
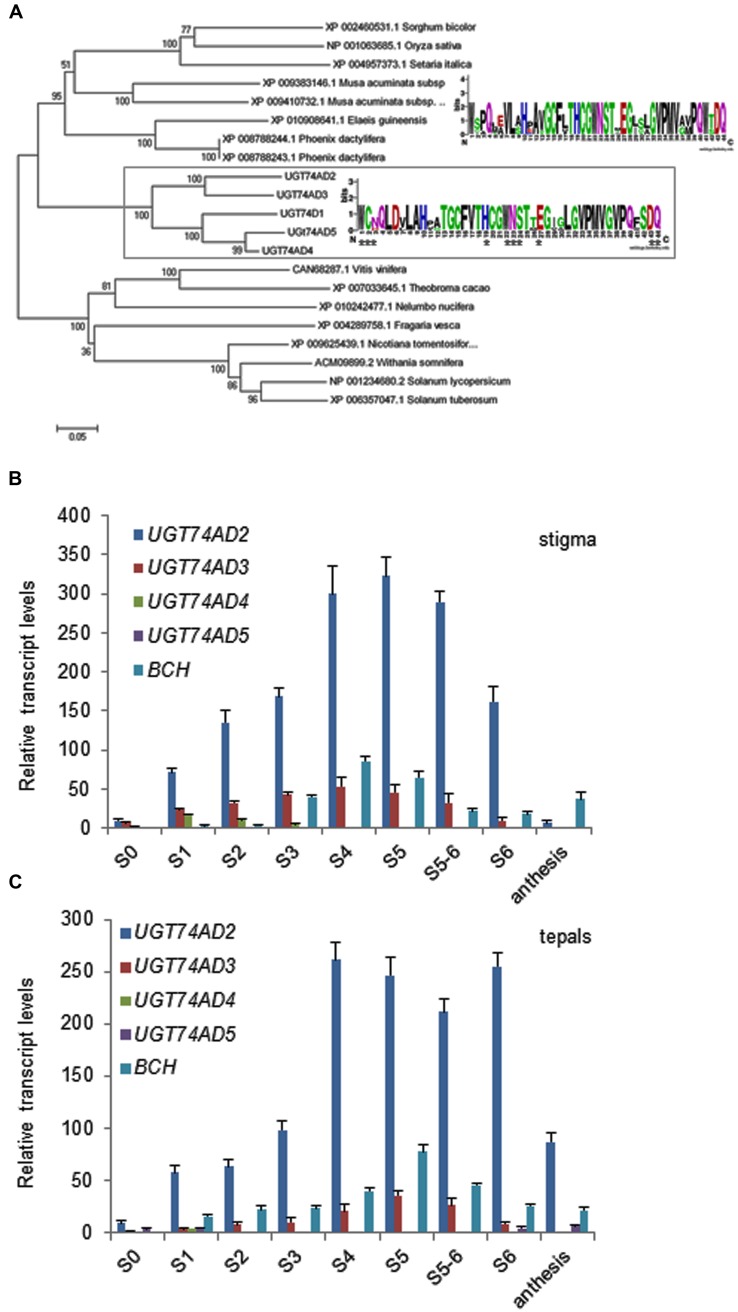
**Analysis of UGT74AD sequences identified in *C. ancyrensis* flowers. (A)** Phylogenetic analysis of UGT74AD proteins. An un-rooted phylogenetic tree was constructed with the neighbor-joining method based on ClustalW multiple alignments. The percentages of replicate trees in which the associated taxa clustered together in the bootstrap test are shown next to the branches. The sequences isolated in this study are framed with UGT74AD1, from *C. sativus*. Inside the frame, the consensus PSPG box from these proteins is shown. The PSPG consensus box for those UGTs more closely related and present as a sister cluster is shown above. **(B)** Expression analysis of *UGT74AD2*, *UGT74AD3*, *UGT74AD4*, and *UGT74AD5* in the stigma during its development. **(C)** Expression analysis of *UGT74AD2*, *UGT74AD3*, *UGT74AD4*, and *UGT74AD5* during the development of tepals. The relative expression levels of *BCH* were incorporated for comparison. Values are means of three technical replicates ±SE, normalized to the internal control gene.

Furthermore, we isolated four genes encoding for glucosyltransferase enzymes with identity to CsUGT2 (UGT74AD1; Supplemenatry Table S3 and Figure S10), involved in crocetin glucosylation in saffron stigma (Moraga et al., 2004). These four enzymes named as UGT74AD2, UGT74AD3, UGT74AD4, and UGT74AD5, showed at their carboxyl terminal end the plant secondary product glycosyltransferase (PSPG) box signature motif, with the conserved residues that interact directly with the UDP-sugar (**Figure 6A**). The phylogenetic analysis showed that the four sequences from *C. ancyrensis*, together with UGT74AD1, are grouped in a well-supported cluster (**Figure 6A**), and separated from those UGT sequences from other plant species that showed the highest identity in the BLAST search analysis. UGT74AD2, UGT74AD3, UGT74AD4, and UGT74AD5 were analyzed for the presence of N-terminal targeting signals or C-terminal membrane anchor signals using ChloroP 1.1 and TargetP v1.1 web-based programs. UGT74AD2 and UGT74AD3 were predicted to have an N-terminal signal peptide for targeting to plastids, as UGT74AD1, while UGT74AD4 and UGT74AD5 were predicted to be cytosolic. For comparative modeling, UGT74AD1, UGT74AD2, UGT74AD3, UGT74AD4, and UGT74AD5 were aligned with MtUGT71G1 and UGT72B1, having their crystal structures available (He et al., 2006; Brazier-Hicks et al., 2007), using the Phyre^2^ server^6^ (Supplementary Figure S10). From this analysis, we observed that UGT74AD2 and UGT74AD3 showed the most similar tridimensional structures compared to UGT74AD1 at the N-terminal and C-terminal domains (Supplementary Figure S10).

Expression analysis of these genes in stigma and tepals showed the highest expression levels for *UGT74AD2* respect to the other *UGTs* (**Figures 6B,C**). *UGT74AD2* expression levels reached its maximum in stigmas at stage S5. In tepals the highest expression of *UGT74AD2* is observed from S4 to S6 and decline thereafter. *UGT74AD3* followed a similar expression pattern compared with *UGT74AD2*, but showed a lower expression levels (**Figures 6B,C**).

## Discussion

Glycosylated apocarotenoids have been analyzed in different plant species (Llanos et al., 2010; Walter et al., 2010), but only in the *Crocus* genus apocarotenoids with many glucose molecules associated to their ends have been detected (Rubio Moraga et al., 2013). Crocins with eight (crocin-1), seven (crocin-2), and six glucose (crocin-3) molecules have been identified in the stigma and tepal of *C. ancyrensis*. The structure of crocin-1 showed a symmetrical structure. The tetrasaccharide present at both ends was *O*-[β-D-Glucopyranosyl)-(1→4)-(β-D-glucopyranosyl)-(1→2)]- *O*-[β-D-glucopyranosyl-(1→6)]-β-D-glucopyranosyl. Crocin-2 showed the same tetrasaccharide at one end, whereas in the other end a trisaccharide was identified as *O*-β-D-glucopyranosyl-(1→2)-*O*-[β-D-glucopyranosyl-(1→6)]-D-glucose. In cell suspension cultures of *C. sativus* a crocetin with two trisaccharide molecules has been identified, two *O*-β-D-glucopyranosyl-(1→2)-*O*-[β-D-glucopyranosyl-(1→6)]-D-glucose groups are present at each end of the molecule (Dufresne et al., 1997), but this crocin has never been reported in the plant. This type of crocin with six glucose molecules, was also identified in *Crocus neapolitanus*, and accordingly the trisaccharide structure designated as neapolitanose (Rychener et al., 1983). Crocin-3 has six glucose molecules and its retention time and spectral data suggest that crocin-3 is a crocetin neapolitanosyl di-ester. The structures of crocin-1, 2, and 3 suggest a sequential addition of glucose molecules over the crocetin skeleton. In fact, crocins with all the different possible substitutions ranging from one to eight glucose units have been identified in stigma and tepal extracts from *C. ancyrensis* (Rubio Moraga et al., 2013). Further, it has been shown that the glucosylation of crocetin into crocin in saffron is sequential (Dufresne et al., 1997). Therefore, the presence of the same crocin structure among different *Crocus* species suggested a similar mechanism involved in crocetin glucosylation. A glucosyltransferase enzyme involved in crocetin glucosylation was isolated from saffron stigmas (Moraga et al., 2004). This enzyme, UGT74AD1, was able to add *in vitro* up to 12 glucose molecules over crocetin. Four UGT74AD1 homologs were isolated from *C. ancyrensis* stigma and tepals, UGT74A2-5. The phylogenetic analysis of all these sequences showed that altogether are grouped in a well supported cluster, separated from UGTs identified in other monocots. Further, the consensus PSPG motif of UGT74AD1-5, compared with the ones identified for other UGTs groups, showed very well conserved amino acid residues that interact directly with the UDP-sugar donor (Caputi et al., 2012); whilst others showed a certain degree of variation allowing the determination of characteristics PSPG box motifs for the different phylogenetic groups. Interestingly, we found that residue at positions 6 (D) was conserved in all the UGT74AD sequences and not present in the other PSPG box from different UGTs clusters (Caputi et al., 2012).

Structural similarity analysis among the isolated UGTs and UGT74AD1 showed that the structures of UGT74AD2 and UGT74AD4 were the most similar ones to UGT74AD1. Furthermore, the predicted location for these proteins was the same than for UGT74AD1, the plastid, in contrast to UGT74AD3 and UGT74AD5, which did not show a plastid location. This plastid location is expected for the glucosylation of crocetin, which formation is catalyzed by CCD2 in the chromoplast (Ahrazem et al., 2015). Expression levels ofUGT74AD2 and UGT74AD4 in tepals and stigma were correlated with the accumulation of crocins in both tissues, suggesting their role in crocin biosynthesis. The accumulation of crocins in stigma began early in the development of the stigma, although the burst of crocins accumulation takes place in the transition from S3 and S4. At this time, the stigma has reached its final size and in the following stages takes place the accumulation of crocins, increasing its coloration. In contrast to stigma, tepals development is delayed, reaching practically the final size at S5, when crocins accumulation was increased. The levels of crocins decreased in both tissues at postanthesis. In saffron it has been observed that reduction of crocins in the stigma at postanthesis is due to an active transport of crocins from the senescent stigma to the new corm that is being developed (Rubio-Moraga et al., 2010) as way of nutrients reutilization from senescent tissues (Guiboileau et al., 2010).

Different mechanisms of regulation have been proposed for carotenoid accumulation in plants, and among them transcriptional regulation of the biosynthetic genes is one of the best characterized in many different plant species (Nisar et al., 2015). However, much less is known about apocarotenoid regulation in chromoplast containing tissues (Castillo et al., 2005; Ahrazem et al., 2010; Gao and Zhu, 2013; Rodrigo et al., 2013). At the earliest developmental stage of stigma and tepals, the low gene expression levels of all carotenogenic genes, were responsible for the low concentration of crocins in both tissues. The expression levels of the upstream genes in the carotenoid pathway, PSY, PDS, Z-ISO, and ZDS, simultaneously increases in the following stages (S1–S2) but not the expression of the downstream genes CRTISO, LCY-II, and BCH, whose expression was increased from S3–S5. PSY is widely recognized as being one of the most important regulatory enzymes in carotenoid biosynthesis (Cazzonelli and Pogson, 2010). Two putative genes encoding for PSY were identified in *C. ancyrensis* flowers, *CaPSY-I* and *CaPSY-II*. Globally, the expression levels of both genes were relatively low compared with other carotenogenic genes acting downstream in the pathway such as *CaZDS*, *CaCRTISO*, *CaLCY-II*, and *CaBCH*. However, it has been shown that PSY protein levels are not equivalently reflected in *PSY* mRNA (Arango et al., 2014) suggesting a complex regulatory system involved in controlling PSY (Kachanovsky et al., 2012; Zhou et al., 2015). Further, several reports showed that *PSY* is a multigene family, and each gene has a tissue-specific expression (Fraser et al., 1999; Shumskaya et al., 2012; Fu et al., 2014; Ampomah-Dwamena et al., 2015). The two *PSY* genes of *C. ancyrensis* were expressed in the stigma and tepal tissues, although at different levels. *CaPSY-II* was expressed in the stigma at higher levels compared with *CaPSY-I* and its expression followed the accumulation of crocins in both tissues, suggesting its involvement in the accumulation of these apocarotenoids in *C. ancyrensis*. Several PSY enzymes have been identified in tomato, tobacco, rice, wheat, citrus, maize, carrot, cassava, loquat, and apple (Busch et al., 2002; Clotault et al., 2008; Welsch et al., 2008, 2010; Howitt et al., 2009; Ampomah-Dwamena et al., 2012, 2015; Kachanovsky et al., 2012; Peng et al., 2013; Fu et al., 2014). In tomato, PSY1 is mainly expressed in fruits and chromoplast-containing tissues, and its expression is developmentally regulated (Fraser et al., 1994; Fantini et al., 2013); PSY2 is predominantly expressed in leaves (Fraser et al., 1999; Giorio et al., 2008) and PSY3 in roots (Fantini et al., 2013). By contrast, with the exception of rice PSY3, expressed in the root (Welsch et al., 2008), the rice PSY1 and PSY2 genes showed quite similar expression patterns and regulation, probably due to the absence of chromoplast-containing tissues in rice. However, the rice PSY1 is mainly responsible for carotenoid supply in chloroplasts (Welsch et al., 2008). Interestingly in maize, PSY1 and PSY2 are both expressed in leaves, but PSY1 is expressed in the endosperm allowing carotenoids accumulation in this tissue (Shumskaya and Wurtzel, 2013). In citrus, PSY1 was highly expressed in fruit peel and flesh (Peng et al., 2013), whereas PSY2 was expressed at low levels. In loquat, with four PSY enzymes, the expression pattern is more complex, PSY1 is responsible for carotenoid synthesis in the fruit peel whereas PSY2A is responsible for carotenoid accumulation in flesh of ripening fruit, whereas PSY2B is expressed in leaves (Fu et al., 2014). The analysis of the phylogenetic relationships and expression patterns among the PSYs of the different plant species, suggests that different PSY genes have been recruited to perform similar roles during the evolution. In particular for carotenoid accumulation in chromoplast, as the tomato PSY1, the PSY1 from citrus, PSY1 and PSY2A from loquat, and this could be as well the case of CaPSY-I for apocarotenoids accumulation in stigma and tepals in *C. ancyrensis*.

Location studies showed that maize PSY2 and PSY3 are localized in plastoglobuli as *Arabidopsis* PSY and rice PSY1, whereas maize PSY1 is localized in a distinct plastid compartment, but not in plastoglobuli (Shumskaya et al., 2012; Shumskaya and Wurtzel, 2013). The hydrophobic analysis of CaPSY-I and CaPSY-II showed the presence of two important hydrophobic domains, similar to the ones predicted for the maize PSY1 enzyme, involved in carotenoid deposition in the seed endosperm (Li et al., 2008). In contrast, maize PSY2 and PSY3, involved in carotenoid biosynthesis in leaves and roots, showed a very different profile, much more hydrophobic, with a putative transmembrane domain, not present in CaPSY-I and CaPSY-II. These differences in the structure of the PSY isoforms could be related to the presence of different plastids with different internal organization (Li and Yuan, 2013), and suggested a differential location of CaPSY-I and II inside the chromoplast.

Phytoene desaturation is catalyzed in two-steps by two phylogenetically related enzymes; PDS and ZDS. Both enzymes are encoded by single genes in tomato, rice, and *Arabidopsis* (Mann et al., 1994; Qin et al., 2007; Chaudhary et al., 2010). However, a small gene family appears to encode PDS and ZDS in some plant species^7^ (Hyun et al., 2012). Five putative *PDS* homologs were identified in stigma and tepals, *CaPDS-I* to *V*. Four out of this five (*CaPDS-I* to *IV*) showed their highest expression levels in the earlier developmental stages (S0–S1) and their expression did not follow crocins accumulation in stigma or tepals, where their showed very reduced expression levels. In contrast, *PDS-V* showed an expression profile more closely related to the levels of crocins in stigma and tepals, suggesting its involvement in the biosynthesis of crocins. Phytoene is the substrate for the isomerase Z-ISO, an integral membrane protein (Chen et al., 2010; Beltran et al., 2015). In stigma and tepals, *CaZISO* reached the highest expression level coincident with the accumulation of crocins in both tissues. ZDS catalysis is performed in conjunction with CRTISO to produce all-*trans*-lycopene. Both, *CaZDS* and *CaCRTISO* expression levels were found to be high in stigmas and tepals. Interestingly, *CaZDS* levels were similar in tepals and stigma, although the levels of crocins in stigmas were much higher than in tepals, suggesting that *CaZDS* is not a limiting enzyme for crocins formation. The product of PDS activity, tetra-*cis*-lycopene, can be transformed to *trans*-lycopene by photoisomerization, but in non-photosynthetic tissues, tetra-*cis*-lycopene is the substrate of the carotenoid isomerase CRTISO (Cazzonelli and Pogson, 2010). Two genes, CCR2/CRTISO1 and CRTISO2, have been proposed to encode CRTISO in *Arabidopsis* (Lange and Ghassemian, 2003), but only CCR2/CRTISO1 has been demonstrated to encode a functional isomerase (Park et al., 2002). Although single copies of both genes are found in other plant species^7^, only one *CRTISO* gene was identified in this study, *CaCRTISO*. This gene is highly expressed in stigma and tepals, and its expression in both tissues is related to the crocin content. It has been suggested that CRTISO has a function controlling the production of apocarotenoids (Cazzonelli and Pogson, 2010) and this seems to be the case for crocetin accumulation in tepals and stigma.

In saffron, two lycopene β-cyclase genes have been isolated (*CstLCY-I* and *CstLCY-II*). CstLCY-II is a chromoplast tissue-specific lycopene β-cyclase, and is one of the key enzymes controlling crocins accumulation in the stigma tissue in saffron (Ahrazem et al., 2010). In stigma and tepals of *C. ancyrensis*, two homologs to *CstLCY-I* and *CstLCY-II* were isolated. The expression of *CaLCY-II* in stigma and tepals of *C. ancyrensis* is also associated with crocins accumulation, and its expression levels clearly differ between both tissues as crocins do, suggesting a clear implication of this enzyme in crocins accumulation. In other plant tissues rich in chromoplast like fruits, the regulation of chromoplast specific lycopene β-cyclase has been shown to be critical for the specific accumulation of carotenoids, like in tomato (Ronen et al., 2000). The pale orange coloration showed by the fruits of the Beta mutant is due to an important increase in the transcription of the chromoplast specific lycopene β-cyclase gene that leads to a higher accumulation of β-carotene. By contrast, the old-gold mutant carries a null allele of this gene resulting in a reduction of β-carotene (Ronen et al., 2000). In pepper, when silencing the *Lcyb* gene, the content of capsanthin decreased significantly (Tian et al., 2014). In *Citrus* species, the up-regulation of the chromoplast specific lycopene β-cyclase genes parallels the massive accumulation of carotenoids accompanying fruit maturation (Rodrigo et al., 2004; Mendes et al., 2011).

β-carotene hydroxylase, was one of the carotenogenic genes showing high levels of expression in stigma and tepals. Only one β-carotene hydroxylase gene was detected in *C. ancyrensis*, while in *C. sativus* two genes have been isolated (Castillo et al., 2005), but only one of them is expressed during the development of the stigma at high levels. Two genes have been also reported in pepper (Bouvier et al., 1998), tomato (Hirschberg, 2001), and Citrus (Kim et al., 2001). In tomato, one gene is expressed in green tissue and the other in flowers (Hirschberg, 2001). In pepper, also one gene is fruit-specific (Borovsky et al., 2013). Interestingly, the β-carotene hydroxylase enzymes from *Crocus* constitute a separate cluster from the other monocots and dicots enzymes. The reason is due to the differences in the N-t part of the protein, probably involved in the association with specific membranous compartments inside the chromoplast (Grilli-Caiola and Canini, 2010) were apocarotenoids are synthesized.

Further, we investigated the expression levels of the gene encoding CaCCD2, which catalyzes the biosynthesis of crocetin dialdehyde from zeaxanthin in *Crocus* species (Frusciante et al., 2014; Ahrazem et al., 2015). In saffron, *CsCCD2* expression follows crocins accumulation during the development of the stigma (Moraga et al., 2009; Frusciante et al., 2014). In *C. ancyrensis*, *CaCCD2* expression levels were also related to the accumulation of crocins in stigma and tepals. In stigma, crocins accumulation begins earlier than in tepals but also declines before. Such pattern is found for *CaCCD2* transcripts when their evolution is compared between stigma and tepals. These data confirm that crocin accumulation in *Crocus* species is mainly controlled at the transcriptional level of *CaCCD2*.

The apocarotenogenesis regulation in stigma and tepals showed that the apocarotenoid levels increased during the development and reached their maximum previous to the anthesis stage. In parallel, transcript levels of the analyzed carotenogenic and *CaCCD2* genes globally increased during stigma and tepal development. Interestingly, the carotenogenic genes encoding for CaPSY-II, CaPDS-V, Ca-ZISO, CaCRTISO, CaLCY-II, and CaBCH, showed a reduction in their expression levels coincident with the highest accumulation of crocins, suggesting a feedback inhibitory mechanism, where certain crocins levels could repress the expression of these genes. A feedback regulatory mechanism has been proposed to act in the carotenoid pathway (Cazzonelli and Pogson, 2010) and apocarotenoids have been recently suggesting to be involved in the control of this pathway (McQuinn et al., 2015).

Overall, apocarotenoid accumulation in stigma and tepals was concomitant with the increase in transcripts levels of carotenogenic and apocarotenogenic genes, thus suggesting that apocarotenoid accumulation during stigma and tepals development may be regulated by gene expression.

## Author Contributions

Oa and LG-G conceived and designed the experiments with the help of AR-M and MJ. LG-G dissected the plant material and performed the RACE-PCR and cloning experiments. AR-M performed HPLC-DAD analysis and contributed to the purification of crocins. MJ performed the 1D and 2D [1H, 1H] (gCOSY and TOCSY and ROESY) and [1H-13C] NMR experiments. OA contributed to the preparation of the RNA samples and performed the qRT-PCR experiments. LG-G wrote the manuscript and all authors contributed to the discussion and approved the final manuscript.

## Conflict of Interest Statement

The authors declare that the research was conducted in the absence of any commercial or financial relationships that could be construed as a potential conflict of interest.

## Footnotes

1 http://www.ncbi.nlm.nih.gov

2 http://www.cbs.dtu.dk

3 http://psort.nibb.ac.jp

4 http://www.clustal.org

5 http://www.megasoftware.net

6 http://www.sbg.bio.ic.ac.uk/phyre2/

7 www.phytozome.net
