# Supplementary material for: Structural characterization of highly glucosylated crocins and regulation of their biosynthesis during flower development in Crocus
Source: Front Plant Sci. 2015 Nov 4;6:971. doi: 10.3389/fpls.2015.00971 (PMC4632010; doi:10.3389/fpls.2015.00971)
Supplement: Supplementary file 1 [file Table_1.DOCX]

Supplemental Table S1. Oligonucleotide sequences used for cloning and gene expression analysis

|  | cDNA amplification | qRT-PCR | RACE-PCR |
| --- | --- | --- | --- |
| *CaPSY* | F: 5'atggcaatagcattacttcg3'  R: 5'tacctaggctttcatcctttc3' | F: 5'gtccagagtccaaagcaac3'  R: 5'agcttggcttagctcagtcagt3' | F: 5' tctcacattacaccttcagct3'  R: 5'cagcctgagccagctcatcct3' |
| *CaPDS-I* | F: 5'ATGAATCTTGTTGGGTTGGT3'  R: 5'ttaggcaatggtcactctggt3' | F: 5'TGATCGTCTGCAGTGGAAAG3'  R: 5'GGCCAAGTCAGCATTTCATT3' | F: 5'ATTGGAGGCGAGAGATGTTCT3'  R: 5'ACTCCTTGCAACCAGCAAGT3' |
| *CaPDS-II* | F: 5'ATGTCGTCGATAAAGGCCAAG3'  R: 5'ctacatgaaaaattcagctag3' | F: 5' AAAGGCCAAGACATCCATTG3'  R: 5'CCGTGTTGTCAAAGTCGATG3' | F: 5'ATCGTGATCTACCTCGCCTTC3'  R: 5'tctcgtagtgtttcaatatga3' |
| *CaPDS-III* | F: 5'ATGAATCTTGTTGGGTTGGT3'  R: 5'tcatctttggttgagggcact3' | F: 5'TGATCGTCTGCAGTGGAAAG3'  R: 5'GGCCAAGTCAGCATTTCATT3' | F: 5'ATTGGAGGCGAGAGATGTTCT3'  R: 5'ACTCCTTGCAACCAGCAAGT3' |
| *CaPDS-IV* | F: 5'ATGTGGGCTTACTCCTCATCT3'  R: 5' tcatctttggttgagggcact 3' | F: 5' AAAGGCCAAGACATCCATTG3'  R: 5'CCGTGTTGTCAAAGTCGATG3' | F: 5'ATCGTGATCTACCTCGCCTTC3'  R: 5'tctcgtagtgtttcaatatga3' |
| *CaPDS-V* | F: 5'atggctcttggtgggttggt3'  R: 5'ttaggcaacaatcatctttg3' | F: 5'ttggaggcgagagatgttct3'  R: 5' ctttccactgcagacgatca3' | F:5'aggcctgaactcgaaagcgcggt3'  R: 5'TGGTGTTTTGACAACATGATAC3' |
| *CaZDS* | F:5'atgatgacgatggctgcttctg3'  R: 5'TCATACAAGGCTAAACTTGTC3' | F: 5'tccctggcccttcacgataa3'  R: 5'ctggcacttgcctcaccaga3' | F: 5'caaggtctccgatatgagcgt3'  R: 5'CTTGTCTGCCAGAGAGAGTTG3' |
| *CaZISO* | F: 5'ATGACGACCTCCCTCCTCCT3'  R: 5'TACCAGTGAAGACCAAAGCT3' | F: 5'ACCATCGGTATGATGGCATT 3'  R: 5'AACCATCTGAGGGTGTCTGG3' | F:5'agaaggtgaggtctccgatgagt3'  R: 5'CGGATAAATTCCTTGTAGAAATC3' |
| *CaLYC-II* | F: 5'ATGATGATCTCTAGTCTTCA3'  R: 5'ctaaacagcttgaagagcca3' | F: 5'gacgagcgaccaaagaagtc3'  R: 5'gaacatcccttcgatttgga3' | F: 5'gcatccgactacgacaccgt3'  R: 5'ACGATATCCAACCTCGACGA3' |
| *CaLYC-I* | F: 5'tggatactcttctaagaactcat3'  R: 5'CTAATCCCTTTCTCGTATTAAG3' | F: 5' gaacatcccttcgatttgga3'  R: 5'gacgagcgaccaaagaagtc3' | F: 5'gtgtaaagatggttcctttaaggt3'  R: 5'AGCTTAGGCAGGATATCCATAC3' |
| *CaBCH* | F: 5'ATGGCGGCCAGAATCTCCC3'  R: 5'tcaggctttgatgtcggagc3' | F: 5'cttgtccgagtggtggatct3'  R: 5'acgacgtcttcgccataatc3' | F: 5'TGCCACGACCCTGGCCGCGTCCT3'  R: 5'TCCTGCTAACTTCCTTCTCCAG3' |
| *CaCrtISO* | F: 5'atgctcagattagggtttcct3'  R: 5'tcatgcaattgtcctgaacct3' | F: 5' ctagggttctggtgctggag3'  R: 5' caactgcttccaatgcttga3' | F: 5'aattcgacgctatcgtgatcgga3'  R: 5'AACACTATGGAGTTTCTGAGGC3' |
| *CaCCD2* |  | F: 5' CTACGTATTCTTAGAGACTAGT 3'  R: 5 ATCAGGACTCTCAAGGCGACA '3' |  |
| *UGT74AD2* | F: 5'caatggagcaaagtcatatcct3'  R: 5'tccctcacttgcaacactcatct3' | F: 5'TCACACAACCTTGTTATCACT3'  R: 5'TCACACAACCTCCACGTCACC3' | F: 5'gtgcatatccgatggattcgact3'  R: 5'AGGGTGGACATGGTGCCGAAGG3' |
| *UGT74AD3* | F: 5'caatggagcaaagtcatatcct3'  R: 5'tccctcacttgcaacactcatct3' | F: 5'CGACTTTGGATCCACCCGAGT3'  R: 5'ACGTTCGGTGCGGCGTTGAAT3' | F: 5'gtgcatatccgatggattcgact3'  R: 5'AGGGTGGACATGGTGCCGAAGG3 |
| *UGT74AD4* | F: 5'TCCAATGGAGCAACAAGGTGAG3'  R: 5'TCACTTGCAACACTCATCTATA3' | F: 5'CTCGACTACGGAGGGGATCG3'  R: 5'ACACTCATCTATAAACTCCAT3' | F: 5' ACTCGTCAACACCCGGTTCCTC3'  R: 5actcacaaccaatcctcgacc'3' |
| *UGT74AD5* | F: 5'TCCAATGGAGCAACAAGGTGAG3'  R: 5'TCATTTGCAACACTGATCAATGA3' | F: 5'CATAGCGCAAACCACACGGCT3'  R: 5'ATCCTTAATACACTTGTCAC3' | F: 5' ACTCGTCAACACCCGGTTCCTC3'  R: 5actcacaaccaatcctcgacc'3' |
