# Supplementary material for: Structural characterization of highly glucosylated crocins and regulation of their biosynthesis during flower development in Crocus
Source: Front Plant Sci. 2015 Nov 4;6:971. doi: 10.3389/fpls.2015.00971 (PMC4632010; doi:10.3389/fpls.2015.00971)
Supplement: Supplementary file 2 [file Table_2.DOCX]

Supplemental Table S2. ^1^H (500 MHz) and ^13^C (125 MHz) NMR spectral data for crocin-1 in D_2_O. Chemical shift (δ, ppm) and coupling constants (J in Hz, in parenthesis)

|  | Atom  number | ^1^H | ^13^C* |  |  |
| --- | --- | --- | --- | --- | --- |
| Crocetin moiety | 1 | - | 167.9 |  |  |
|  | 2 | - | 124.2 |  |  |
|  | 3/3’ | 7.35 | 142.1 |  |  |
|  | 4/4’ | 6.59 | 124.2 |  |  |
|  | 5/5’ | 6.71 | 145.8 |  |  |
|  | 6 | - | 137.9 |  |  |
|  | 7/7’ | 6.41 | 135.6 |  |  |
|  | 8/8’ | 6.76 | 132.0 |  |  |
|  | Me-9/Me-9’ | 1.88 | 11.8 |  |  |
|  | Me-10/Me-10’ | 1.87 | 11.8 |  |  |
|  |  |  |  |  |  |
| Carbohydrate moiety |  |  |  |  |  |
| Moiety A | 1 | 5.66 | 93.0 |  |  |
|  | 2 | 3.72 | 80.9 |  |  |
|  | 3 | 3.64 | 74.3 |  |  |
|  | 4 | 3.47 | 70.1 |  |  |
|  | 5 | 3.64 | 74.7 |  |  |
|  | 6 | 4.03, 3.72 | 67.8 |  |  |
|  |  |  |  |  |  |
| Moiety B | 1 | 4.57 | 103.0 |  |  |
|  | 2 | 3.16 | 73.1 |  |  |
|  | 3 | 3.47 | 73.6 |  |  |
|  | 4 | 3.47 | 77.8 |  |  |
|  | 5 | 3.23 | 73.1 |  |  |
|  | 6 | 3.55, 3.50 | 59.3 |  |  |
|  |  |  |  |  |  |
|  | 1 | 4.30 | 102.4 |  |  |
| Moiety C | 2 | 3.09 | 72.9 |  |  |
|  | 3 | 3.29 | 75.4 |  |  |
|  | 4 | 3.21 | 69.4 |  |  |
|  | 5 | 3.26 | 75.7 |  |  |
|  | 6 | 3.71, 3.55 | 60.4 |  |  |
|  |  |  |  |  |  |
| Moiety D | 1 | 4.32 | 102.2 |  |  |
|  | 2 | 3.13 | 73.2 |  |  |
|  | 3 | 3.31 | 75.4 |  |  |
|  | 4 | 3.21 | 69.4 |  |  |
|  | 5 | 3.25 | 75.7 |  |  |
|  | 6 | 3.73, 3.54 | 60.4 |  |  |

*Values measured from gHSQC and gHMBC experiment
