# Supplementary material for: Structural characterization of highly glucosylated crocins and regulation of their biosynthesis during flower development in Crocus
Source: Front Plant Sci. 2015 Nov 4;6:971. doi: 10.3389/fpls.2015.00971 (PMC4632010; doi:10.3389/fpls.2015.00971)
Supplement: Supplementary file 3 [file Table_3.DOCX]

Supplemental Table S3. Proteins involved in crocins biosynthesis in *C. ancyrensis* stigma and tepals

| Protein | Accession number | Amino acids | MW (kDa) | % identity with GenBank sequences |
| --- | --- | --- | --- | --- |
| CaPSY-I | KT124375 | 421 | 47.54 | 81% AFC95818.1 |
| CaPSY-II |  | 387 | 43.89 | 72% AFP35592 |
| CaPDS-I | KT124376 | 527 | 59.01 | 97% AAO24235.1 |
| CaPDS-II | KT124377 | 429 | 48.68 | 72% XP_010918129.1 |
| CaPDS-III | KT124378 | 565 | 62.98 | 97% AAO24235.1 |
| CaPDS-IV | KT124379 | 527 | 59.96 | 69% XP_010918125.1 |
| CaPDS-V | KT124380 | 565 | 62.91 | 96% AAO24235.1 |
| CaZISO | KT124385 | 360 | 40.30 | 74% XP_010913064.1 |
| CaZDS | KT124386 | 561 | 62.12 | 89% AFH53814.1 |
| CaCrtISO | KT124381 | 590 | 65.06 | 78% XP_009415303.1 |
| CaLCY-1 | KT124384 | 501 | 56.62 | 90% ADA82242.1 |
| CaLCY-2 | KT124383 | 474 | 52.80 | 90% ADA82241.1\| |
| CaBCH | KT124382 | 304 | 33.74 | 86% CAC95130.2 |
| UGT74AD2 | KT728827 | 450 | 50.22 | 63% AAP94878 |
| UGT74AD3 | KT728828 | 451 | 50.39 | 64% AAP94878 |
| UGT74AD4 | KT728829 | 458 | 50.87 | 73% AAP94878 |
| UGT74AD5 | KT728830 | 459 | 51.13 | 75% AAP94878 |
