# Supplementary material for: Structural characterization of highly glucosylated crocins and regulation of their biosynthesis during flower development in Crocus
Source: Front Plant Sci. 2015 Nov 4;6:971. doi: 10.3389/fpls.2015.00971 (PMC4632010; doi:10.3389/fpls.2015.00971)
Supplement: Supplementary file 4 [file Image_1.PDF]

Supplemental Figure S1

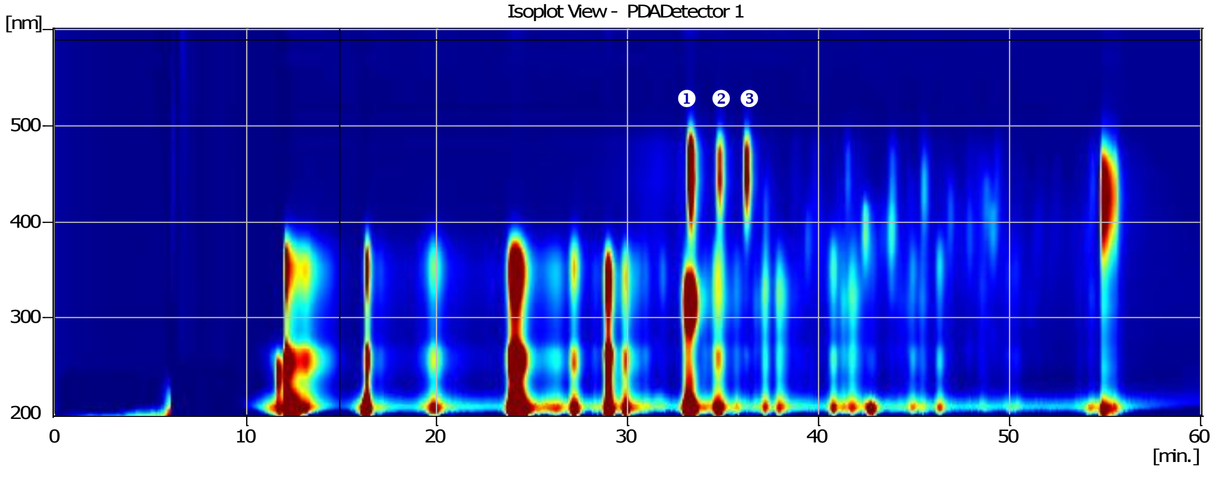

Supplemental Figure S1. HPLC-PDA/UV isoplot chromatogram showing all the compounds detected (200-550 nm) of stigma aqueous extracts of *Crocus ancyrensis*. The numbers correspond to crocin-1 (1), crocin-2 (2) and crocin-3 (3).

Supplemental Figure S2

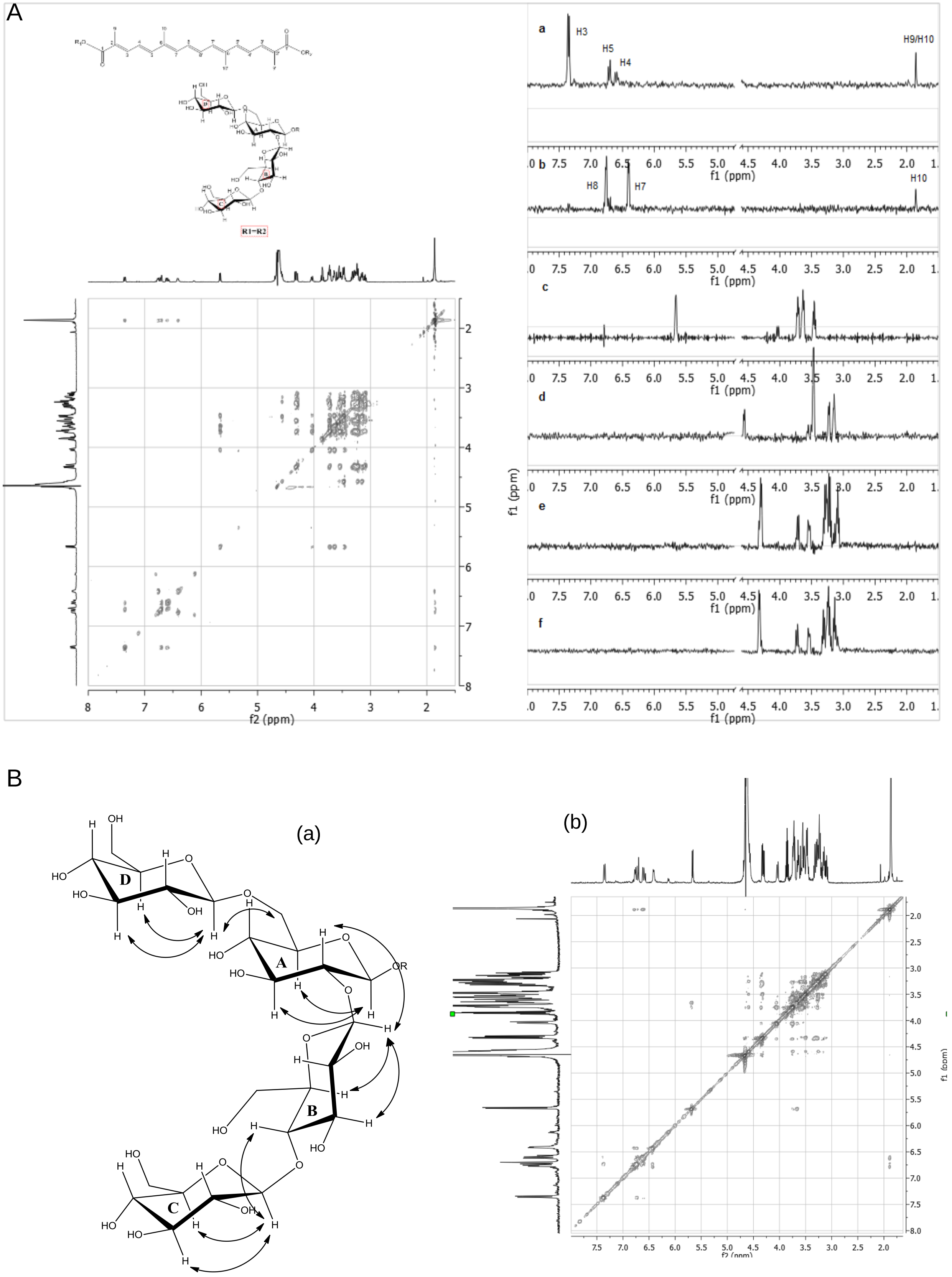

Supplemental Figure S2. A. TOCSY spectrum and relevant traces showing correlations corresponding to a) H3 (7.35), (b) H7 (6.41), (c) H1-A (5.66), (d) H1-B (4.57), (e) H1-C (4.30), and (f) H1-D (4.32 ppm). (B) ROESY experiment of crocin-1. (a) Relevant ROESY correlations for the glycosyl moieties of crocin-1. (b) Contour plot.

Supplemental Figure S3

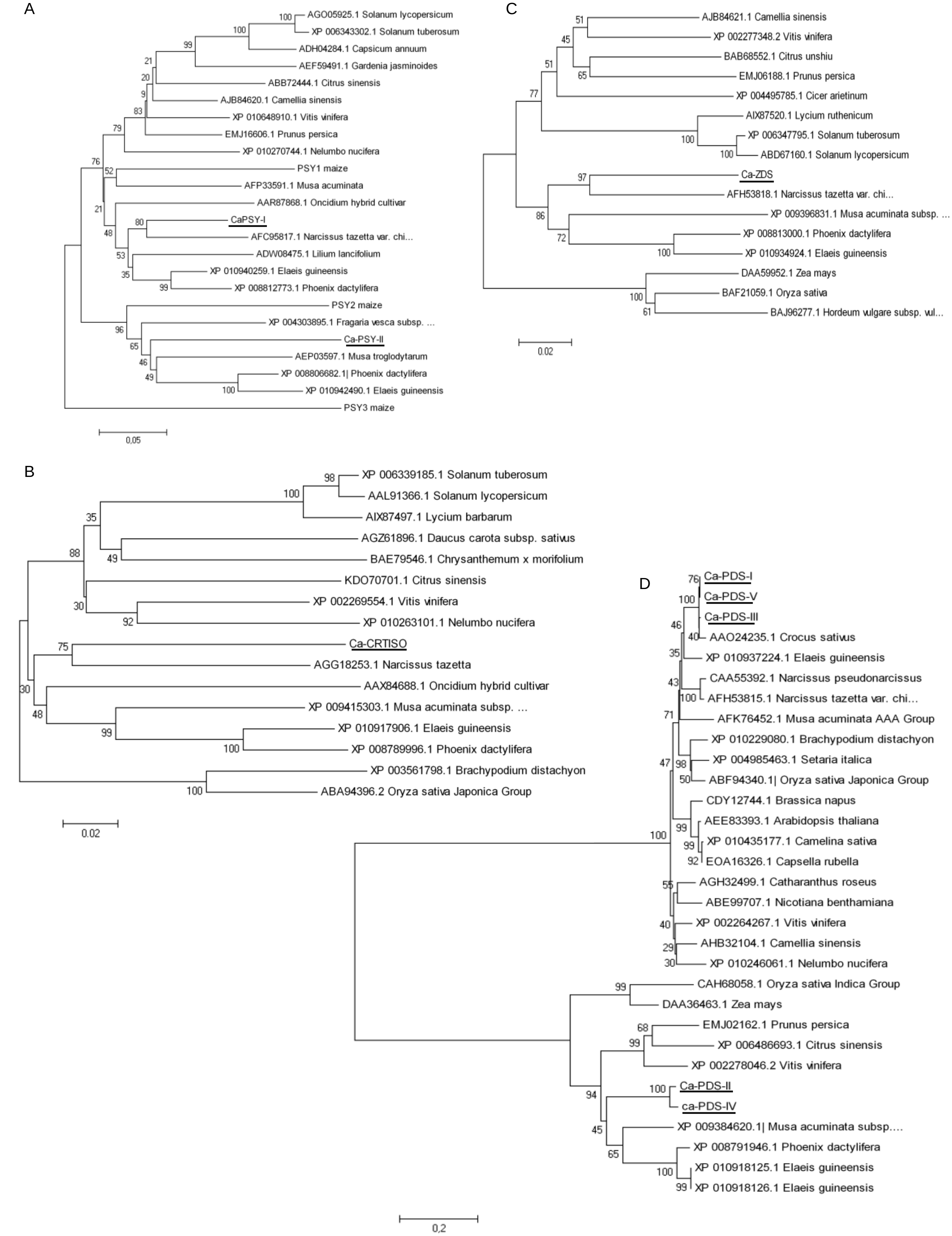

Supplemental Figure S3. Phylogenetic trees (neighbor-joining) of carotenogenic enzymes isolated *from Crocus ancyrensis* stigma and tepals. Underlined are the carotenogenic enzymes isolated in this study. A, Phytoene synthase enzymes (PSY). B, Carotene isomerase enzymes (CtrISO) .C, δ-carotene desaturase enzymes (ZDS). D, Phytoene desaturase enzymes (PDS). Numbers associated with the branches are the neighbor-joining bootstrap values. The length of branch expresses evolutionary distance, with its scale being 0.05.

Supplemental Figure S4

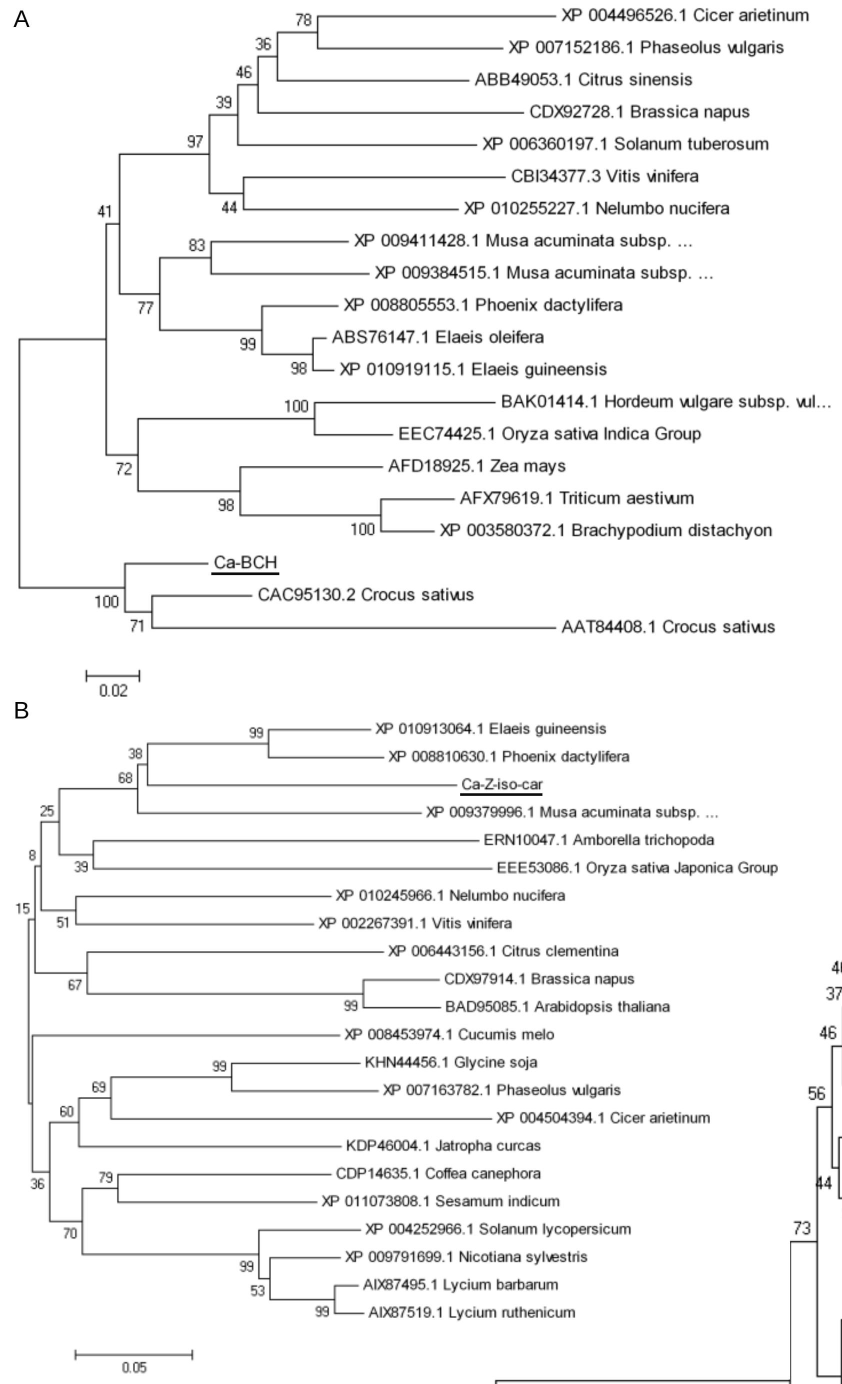

C

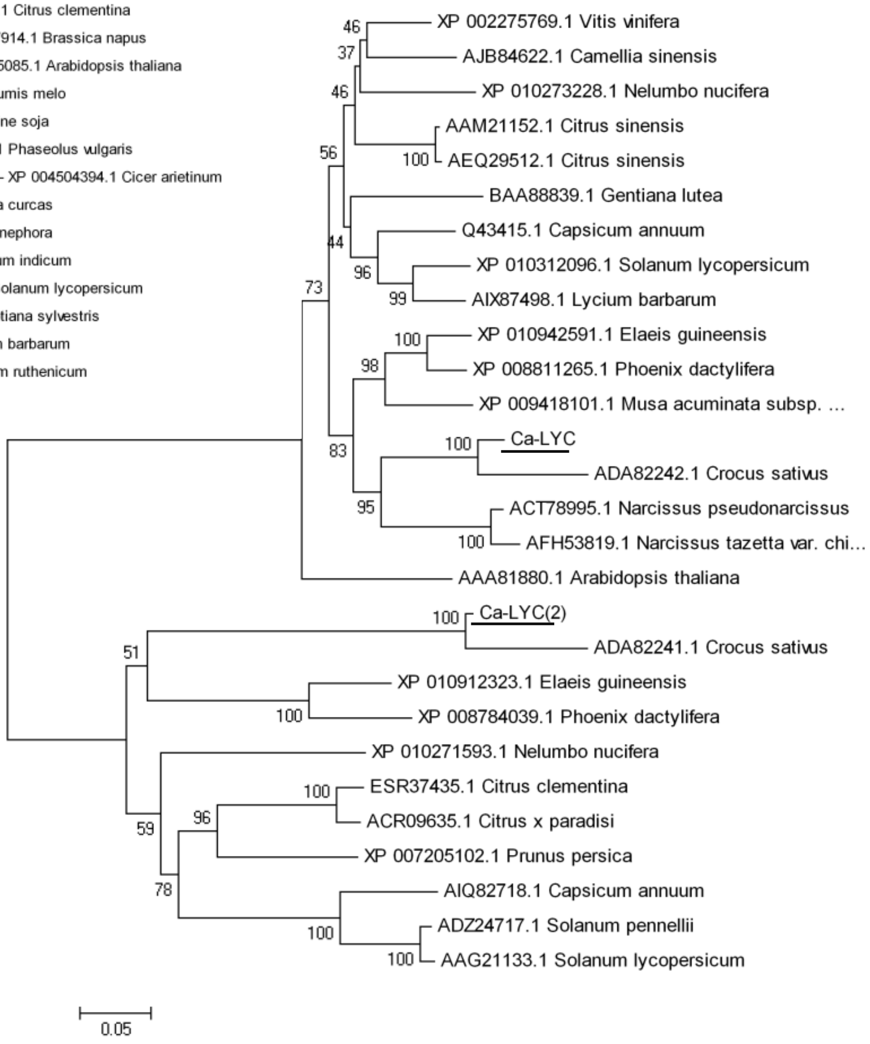

Supplemental Figure S4. Phylogenetic trees (neighbor-joining) of carotenogenic enzymes isolated from *Crocus ancyrensis* stigma and tepals. Underlined are the carotenogenic enzymes isolated in this study. A, β-carotene hydroxylase enzymes (BCH). B15-cis-δ-carotene isomerase enzymes (ZISO). C, Lycopene β-cyclase enzymes (LCY). Numbers associated with the branches are the neighbor-joining bootstrap values. The length of branch expresses evolutionary distance, with its scale being 0.05.

Supplemental Figure S5

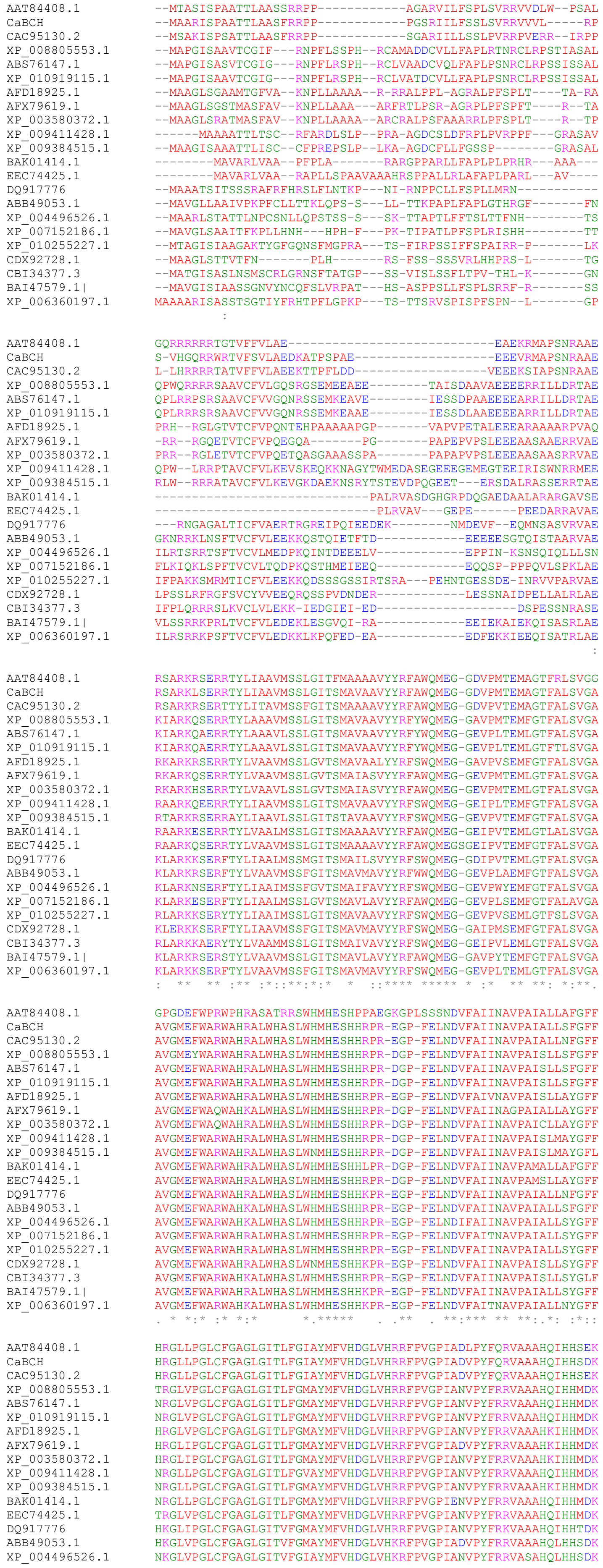

Supplemental Figure S5. Multiple sequence alignment of β-carotene hydroxylase (BCH) amino acid sequences.

Supplemental Figure S6

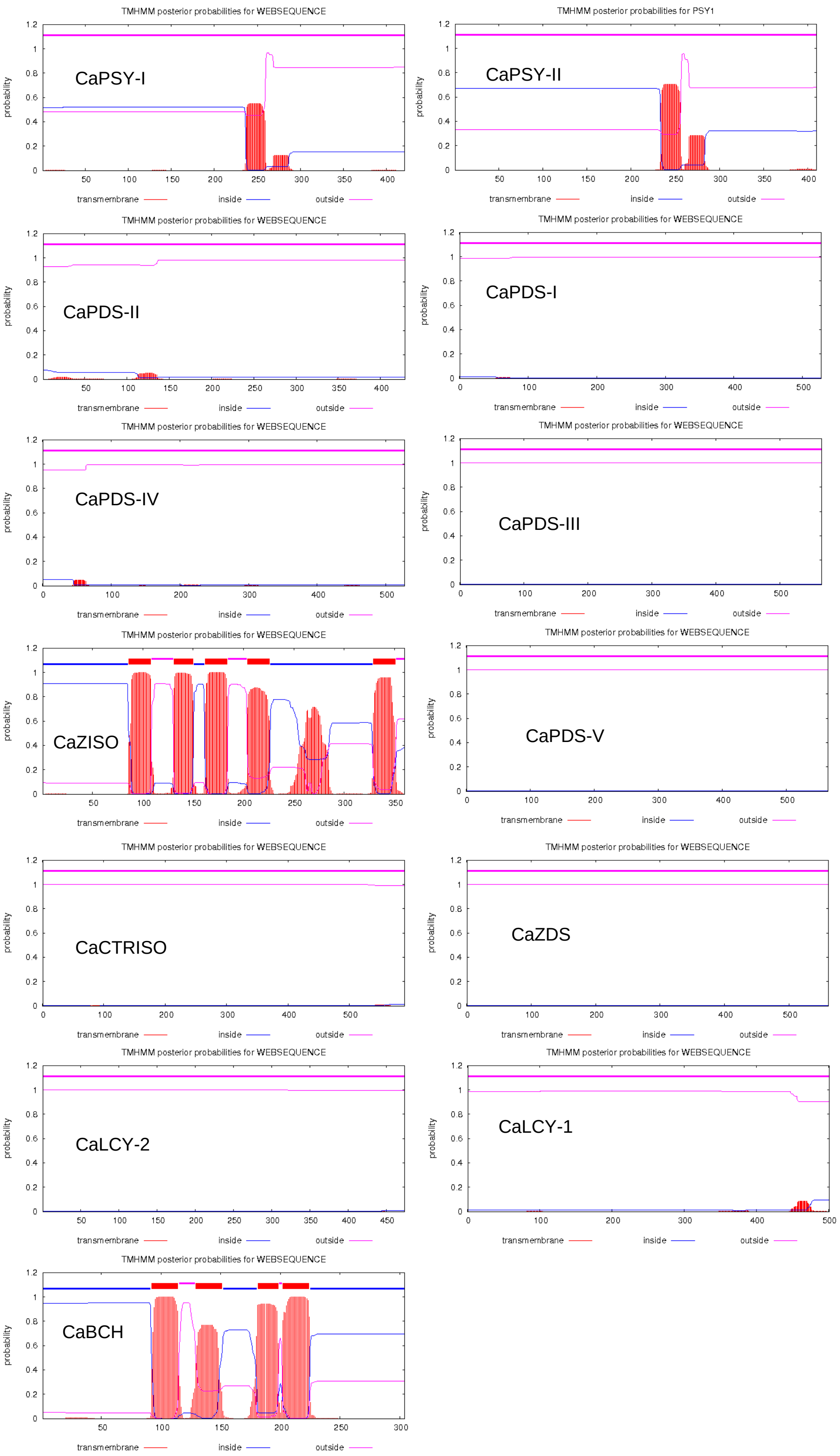

Supplemental Figure S6. Putative hydrophobic of *Crocus ancyrensis* carotenogenic enzymes, evaluated by the TMHMM Server v. 2.0.

Supplemental Figure S7

A

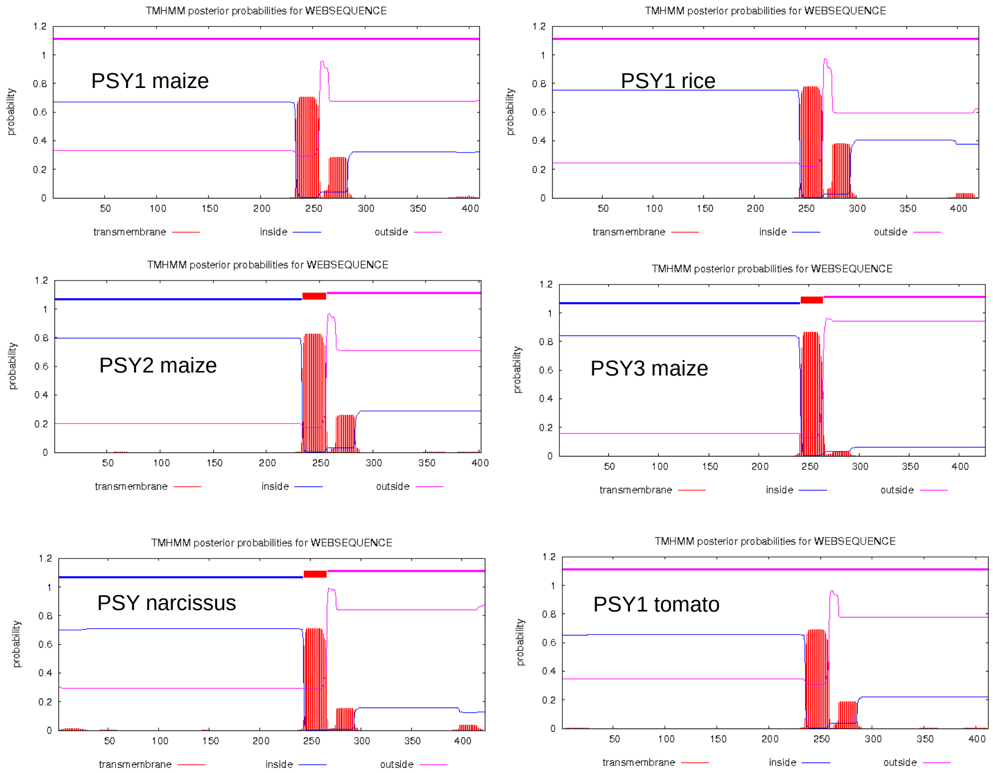

B

```
PSY3              MMSTSRAVKSPACAARRRQWSADAPNRTATFLACRHG-RRLGGGGGAPCSVRA-------
PSY2              MAAGS-----------SAVWA-------AQHPACSGGKFHHLSPSHSHCRPRRAL-----
CaPSY-II          MSGSAV----------LLVSS---PGETTMSSSFSHH---FIGRNNASCKIRSKRVLKSR
PSY1              -MAII-----------LVRAA---SPGLSAADSISHQ-GTLQCS----TLLKTKRPAARR
rice              MAAITL----------LRSAS---LPGLSDALARDAAAVQHVCSS---CLPSNNKEKKRR
CaPSY             -MAIAL----------LRVFS---PIEFPIGLGIEGD---LRGKK--YDGSLKR--KKQI
PSY               -MVVAI----------LRVVS---PIEIPIGLGLSEANWRFSGPK--YDNLHRKKSRLSV
                                      :           .

PSY3              ---EGSNTIVCLEAEAWGGAPA----LPGLRVAAPSPGDAFVVPSEQRVHEVVLRQAALA
PSY2              ------------QTPPALPARRSGASPPRASLAAAAPAVAVRTASEEAVYEVVLRQAALV
CaPSY-II          -------------------QIS-SKVLTFSSILVT----NNPRTSEDIVYEVIVKQATLV
PSY1              WM-PCSLL----GLHPW--EAGRPSPAVYSSLAVNPAGEA-VVSSEQKVYDVVLKQAALL
rice              WI-LCSLKYACLGVDPAPGEIAR-TSPVYSSLTVTPAGEA-VISSEQKVYDVVLKQAALL
CaPSY             WKMYSLSKYACVGLDPEGNG----NFMIRSSVVASPAEE--FISSEQRVYDVVLKQAALV
PSY               YSLYTTSKYACVGFEPESNG----KFQIRSSLVANPAGEATISSSEQKVYDVVLKQAALV
                                                 : .          **: *::*:::**:*

PSY3              AAAPRTARI------------EPVPLDGGLKAAFHRCGEVCREYAKTFYLATQLMTPERR
PSY2              EAATPQRRRTRQPRWAEEEEEERVLGWGLLGDAYDRCGEVCAEYAKTFYLGTQLMTPERR
CaPSY-II          REQRTRA-------------SVVPKDRNLLNEAYGRCEEVCAEYAKTFYLGTLLMTPERQ
PSY1              KRQLRTP--VLD---ARPQDMDMP--RNGLKEAYDRCGEICEEYAKTFYLGTMLMTEERR
rice              KRHLRPQPHTIP---IVPKDLDLP--RNGLKQAYHRCGEICEEYAKTFYLGTMLMTEDRR
CaPSY             REQGRI-TTPID---VKPD-MAVPGTLYLLKEAYDRCGEVCAEYAKTFYLGTLLMTPERR
PSY               KDQTKSSRKSTD---VKPD-IVLPGTVYLLKDAYDRCGEVCAEYAKTFYLGTLLMTPERR
                                              *  *: ** *:* ********.* *** :*:

PSY3              IAIWAIYVWCRRTDELVDGPNASHISALALDRWESRLEDIFAGRPYDMLDAALSDTVARF
PSY2              KAVWAIYVWCRRTDELVDGPNASYITPTALDRWEKRLEDLFEGRPYDMYDAALSDTVSKF
CaPSY-II          RAIWAIYVWCRRTDELVDGPNASYMTPTALDRWEKRLYDLFEGHPYDLYDAALSDTINKF
PSY1              RAIWAIYVWCRRTDELVDGPNANYITPTALDRWEKRLEDLFTGRPYDMLDAALSDTISRF
rice              RAIWAIYVWCRRTDELVDGPNASHITPSALDRWEKRLDDLFTGRPYDMLDAALSDTISKS
CaPSY             RAIWAIYVWCRRTDELVDGPNASHITPSALDRWESRLEDLFAGRPYDMFDAALSDTVSQF
PSY               RAIWAIYVWCRRTDELVDGHNASHITPSALDRWEARLEDLFDGRPYDMFDAALSDTVSRF
                   *:**************** **.:::  ****** ** *:* *:***: *******: :

PSY3              PVDIQPFRDMIEGMRMDLKKSRYRSFDELYLYCYYVAGTVGLMSVPVMGISPASRAATET
PSY2              PVDIQPFKDMVQGMRLDLWKSRYMTFDELYLYCYYVAGTVGLMTVPVMGIAPDSKASTES
CaPSY-II          PIDIQPFKDMIEGMRSDMKKSRYKNFDELYLYCYYVAGTVGLMSVPVMGISPNSKASAED
PSY1              PIDIQPFRDMIEGMRSDLRKTRYNNFDELYMYCYYVAGTVGLMSVPVMGIATESKATTES
rice              PIDIQPFRDMIEGMRSDLRKTRYKNFDELYMYCYYVAGTVGLMSVPVMGIAPESKATTES
CaPSY             PVDIQPFKDMVEGMRLDLKKSRYKNFDELYLYCYYVAGTVGLMSVPVMGIAPESKATTES
PSY               PVDIQPFMDMVEGMRMDLKKSRYKNFDELYLYCYYVAGTVGLMSVPVMGIAPESLAEAES
                  *:***** **::*** *: *:** .*****:***********:******:  * * :*

PSY3              VYKGALALGLANQLTNILRDVGEDARRGRIYLPQDELEMAGLSDADVLDGRVTDEWRGFM
PSY2              VYNAALALGIANQLTNILRDVGEDARRGRIYLPLDELAQAGLTEEDIFRGKVTGKWRRFM
CaPSY-II          VYNSALALGIANQLTNILRDIGEDARRGRIYLPQDELAQAGLSDDDIFRGRVTDKWRNFM
PSY1              VYSAALALGIANQLTNILRDVGEDARRGRIYLPQDELAQAGLSDEDIFKGVVTNRWRNFM
rice              VYSAALALGIANQLTNILRDVGEDARRGRIYLPQDELAEAGLSDEDIFNGVVTNKWRSFM
CaPSY             VYNAALALGIANQLTNILRDVGEDARRGRIYLPQDELAQAGLSDEDVFSGKVTDKWRNFM
PSY               VYNAALALGIANQLTNILRDVGEDARRGRIYLPQDELAEAGFSDEDVFTGQVTDKWRSFM
                  **..*****:*********:*********** ***  **::: *:: * ** .** **

PSY3              RGQIARARAFFRQAEEGATELNQESRWPVWSSLLLYRQILDEIEANDYDNFTRRAYVPKT
PSY2              KGQIQRARLFFDEAEKGVTHLDSASRWPVLASLWLYRQILDAIEANDYNNFTKRAYVGKA
CaPSY-II          KGQITRARMFFEEAEKGITELNSASRWPVLASLLLYRQILDAIEANDYNNFTKRAYVGKA
PSY1              KRQIKRARMFFEEAERGVNELSQASRWPVWASLLLYRQILDEIEANDYNNFTKRAYVGKG
rice              KRQIKRARMFFEEAERGVTELSQASRWPVWASLLLYRQILDEIEANDYNNFTKRTYVGKA
CaPSY             KNQIKRARMFFQEAEKGVTELSQASRWPVLASLLLYRQILDEIEKNDYNNFTKRAYVSKV
PSY               KRQIKRARMFFEQAEKGVTELSQASRWPVWASLLLYRQILDEIEANDYNNFTKRAYVSKV
                  : ** *** ** :**.* ..*.. ***** :** ******* ** ***:***:*:** *

PSY3              KKLMALPKAYLRSLVVPSSSSQAESRRRYSTLT
PSY2              KKLLSLPLAYARAAVAP----------------
CaPSY-II          KKLVSLPLAYARAFAGPSKV-------------
PSY1              KKLLALPVAYGKSLLLPCSLRNGQT--------
rice              KKLLALPVAYGRSLLMPYSLRNSQK--------
CaPSY             KKLAALPIAYGRALISPASTQRQSSRDSSLLKG
PSY               KRLAALPLAYGKSLLIPLSLRSSLSKA------
                  *:* :** ** ::   *
```

C

Maize PSY3	100.00	64.75	62.77	63.40	62.66	63.77	63.43
Maize PSY2	64.75	100.00	68.98	65.03	65.56	68.75	66.24
CaPSY-II	62.77	68.98	100.00	67.46	67.11	70.74	68.24
Maize PSY1	63.40	65.03	67.46	100.00	81.91	71.18	70.37
Rice PSY1	62.66	65.56	67.11	81.91	100.00	70.90	69.95
CaPSY-I	63.77	68.75	70.74	71.18	70.90	100.00	79.76
Narcissus PSY	63.43	66.24	68.24	70.37	69.95	79.76	100.00

Supplemental Figure S7. A) Putative hydrophobic profiles of *different PSY* enzymes, evaluated by the TMHMM Server v. 2.0. B) Multiple sequence alignment of the PSY amino acid sequences. C) Ideentity of different PSY enzymes

Supplemental Figure S8

	1	2	3	4	5
1: CaPDS-III	100.00	98.86	97.70	18.45	20.89
2: CaPDS-I	98.86	100.00	99.05	18.45	20.80
3: CaPDS-V	97.70	99.05	100.00	18.45	20.89
4: CaPDS-II	18.45	18.45	18.45	100.00	94.41
5: caPDS-IV	20.89	20.80	20.89	94.41	100.00

Supplementary Figure S8. Percent Identity Matrix on CaPDS amino acid sequences created by Clustal2.1.

Supplemental Figure S9

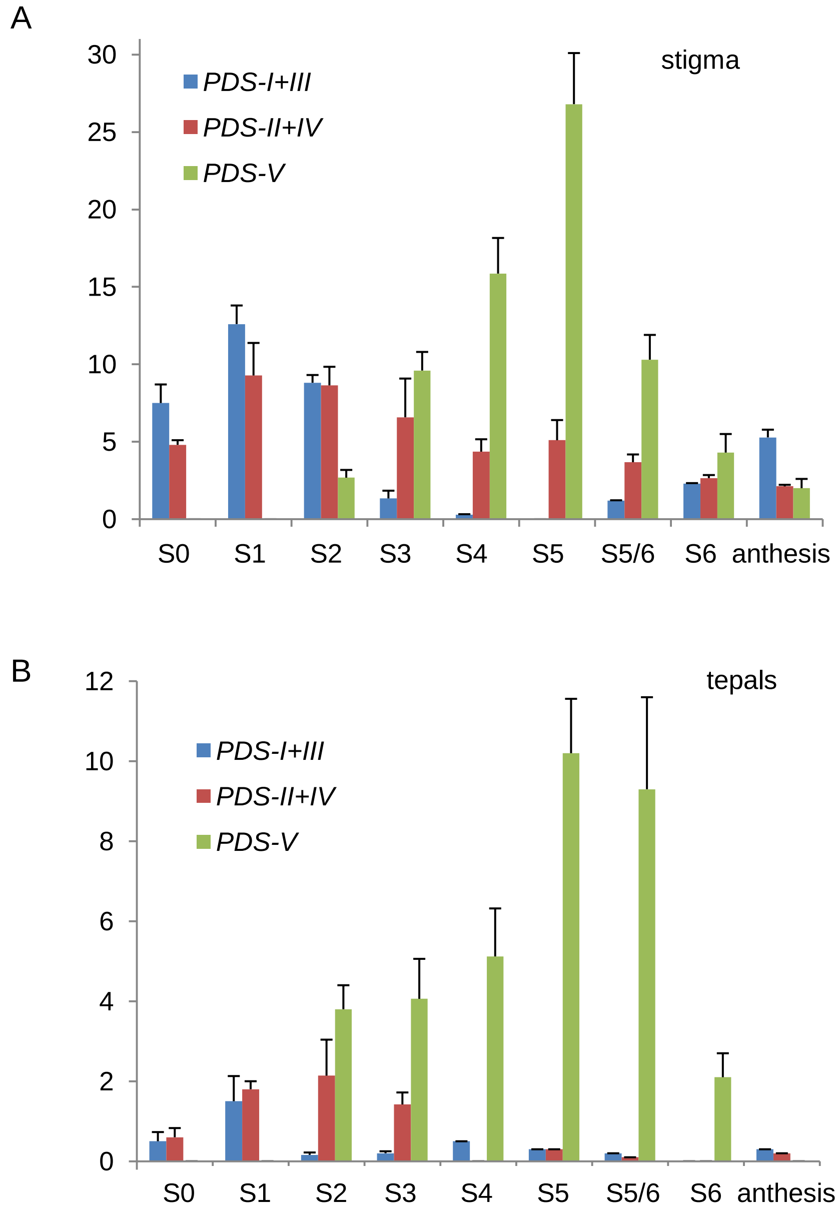

Supplemental Figure S9. *CaPDS* (I-V) expression analyses during stigma and tepal development in flowers of *C. ancyrensis*. (A) Expression levels in stigma tissue. (B) Expression levels in tepals

Supplemental Figure S10

A

```
UGT74AD2      -------MEQSHILLLPFPSQSHVNPMLQFGKRLASHNLVITLASTKFLTNLAKPESGPI
UGT74AD3      -------MEQSHILLLPFPSQSHVNPMLQFGKRLASHNLHVTLANTKFLNNTTKPDPGPV
UGT74D1       MEQKDVNGNKCHILLLPCPAQGHINPILQFGKRLASHNLLTTLVNTRFLSNSTKSEPGPV
UGT74AD5      MEQQGEIGKKCHILLLPYPAQGHINPLLQFGKRLAYHNLDTTLVNTRFLSNSTKPEPGPV
UGT74AD4      MEQQGEIGKKCHILLLPYPAQGHINPLLQFGKRLAYHNLDTTLVNTRFLSNSTKPEPGPV
                      ::.****** *:*.*:**:******** ***  **..*:**.* :* : **:

UGT74AD2      RIECISDGFDSGGSKVAPNLRAYLDRLESVGSETLSDLIESLESKGRPVHVLVYDPFLAW
UGT74AD3      SIESISDGFDSDGFNAAPNVESYLDRLESVGSETLSDLIESLKSKSRPVHVLVYDPFLAW
UGT74D1       NIECISDGFDSGGMNAAPSRRAYFDRLESVGSETLFGLIESLRSRGRPAHVLVYDPFLPW
UGT74AD5      NIECISDGFDLGGMNAAASRQAYFDRLESVGSETLSDLIESLRSRGRPAHVLVYDPFLPW
UGT74AD4      NIECISDGFDLGGMNAAASRQAYFDRLESVGSETLSDLIESLRSRGRPAHVLVYDPFLPW
               **.******  * :.* . .:*:**********  *****.*:.**.********* *

UGT74AD2      GWEVANRFGLRSAAFFTQSCAVDVIYGHLWEGRVKLPVT-EPVRLPGLGPLEPRDLPSYL
UGT74AD3      AWDVADRFGLRSAAFFTQSCAVDVIYGHVWEGWVEVPVT-DRVRLPGLGPLEPRDLPSYV
UGT74D1       AMNVAERSGLRSVAFFTQPCAVDTIYRHVWEGRIKVPVT-EPVRLPGLPPLEPSDLPSFV
UGT74AD5      AADVAERSGLRSVAFFTQACAVDIIYRHVWDRQIEPPVTTGPVRFPGLPPLEPSDLPSFV
UGT74AD4      AADVAERSGLRSVAFFTQACAVDIIYRHVWDRQIEPPVTTGPVRFPGLPPLEPSDLPSFV
              . :**:* ****.***** **** ** *:*:  :: ***   **:*** **** ****::

UGT74AD2      TETW-ATSCPDFFEIIVNQYKNLEKADAVLINSFYELEKEEFDWMKSQL--PVKSIGLTI
UGT74AD3      SDSDRVPKYPGYAALLMNQYKNLEKVDVVLVNSFYELEKKEFDWMKSRL--PVKSIGPTV
UGT74D1       TDSDPV-INPDLLPLLVNQHKNLDKADMVLINSIYELEHEEFDWMESRLPLPVKSIGPTV
UGT74AD5      TGSDPV-VNPDLLSLVVNQYKNLEKADMVLINSIYELEHEEFDWMKSRL--PVKSIGPTV
UGT74AD4      TGSDPV-VNPDLLSLVVNQYKNLEKADMVLINSIYELEHEEFDWMKSRL--PVKSIGPTV
              : :  .   *    :::**:***:*.* **:**:****::*****:*:*  ****** *:

UGT74AD2      PSTYLDNRIPSDSYYVFDTHTIDIAPYIDFLDSNPPSSVIYVAFGTMSTLSPEQANEIAT
UGT74AD3      PSTYLDNRIPSDSYYVFDTHTIDIAPYIDFLDSNPPRSVIYVSFGSMAAPSPEQTQEIAS
UGT74D1       PSTYLDNRIPSDSHYGFNLYTPDTTPYLDWLDSKAPNSVIYVSFGSLSSLSPDQTNEIAS
UGT74AD5      PSTYLDNRIPWDSHYGFNIHTLDTAHYMEWLDSKPRDSVVYVSFGSMASLSPDQTDAVAS
UGT74AD4      PSTYLDNRIPWDSHYGFNIHTLDTAHYMEWLDSKPRDSVVYVSFGSMASLSPDQTDAVAS
              ********** **:* *: :* * : *:::***:   **:**:**:::: **:*:: :*:

UGT74AD2      GLIASEKRFIWVVRASELPKLPNTFIEDTTDRGMLVTWCNQLDVLAHPATGCFVTHCGWN
UGT74AD3      AFVATKKCFIWVVRKSELAKLPKEFVRETAGRGQVVTWCNQLDVLAHVGTGCFVTHCGWN
UGT74D1       GLIATNKSFIWVVRTSELAKLPANFTQENASRGLVVTWCDQLDVLAHVATGCFVTHCGWN
UGT74AD5      GLIATKKSFIWVVRTSELAKVPADFIRESAGRGLVVSWCNQLDVLAHVATGCFVTHCGWN
UGT74AD4      GLIATKKSFIWVVRTSELAKVPADFIRESAGRGLVVSWCNQLDVLAHPATGCFVTHCGWN
              .::*::* ****** *** *:*  * .:.: ** :*:**:******* .***********

UGT74AD2      STTEGIGLGVPMVGVPQFSDQTTNAKYVEDVWKVGVRVKT-DEKGFVRGEELERCIKEVM
UGT74AD3      STTEGIGLGVPMVGVPQFSDQTTNAKYVEDVWKVGVRVKT-DEKGFVRGEELERCIKEVM
UGT74D1       STMEGIALGVPMVGVPQWSDQPMNAKYVEDVWKVGVRAKT-YGKDFVRGEEFKRCVEEVM
UGT74AD5      STMESIAFGVPMVGVPQWSDQPTVVKYVEDVWKVGVRARKIDGEDFVRGEEFKRCVEEVM
UGT74AD4      STTEGIGLGVPMVGVPQFSDQTTNAKYVEDVWKVGVRVK-TDEKGFVRGEELERCIKEVM
              ** *.*.:*********:***   .***********.:    : *****::**::***

UGT74AD2      DGERSGEIRENAKKWSELAKEAISEGGSSDKSIMEFIDECCK
UGT74AD3      DGERSGEIRENAKKWSELAKEAISEGGSSDKSIMEFIDECCK
UGT74D1       DGERSGKIRENAARWCKLAKDSVSEGGSSDKCIKEFIHQCCK
UGT74AD5      DGERSGEIRENAAKWSKMAKESVSEGGSSDKCIKDFIDQCCK
UGT74AD4      DGERSGEIRENAKKWSELAKEAISEGGSSDKSIMEFIDECCK
              ******:***** :*.::**:::*******.* :**.:***
```

B

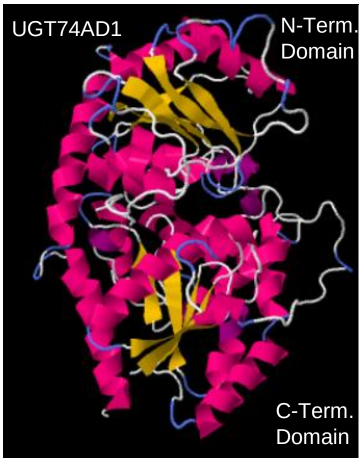

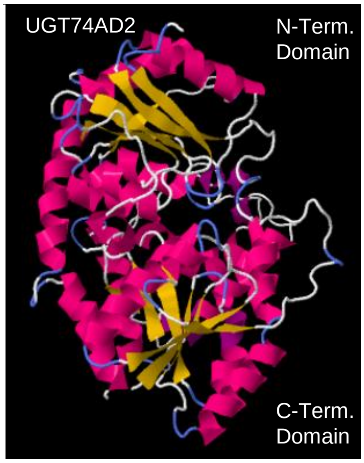

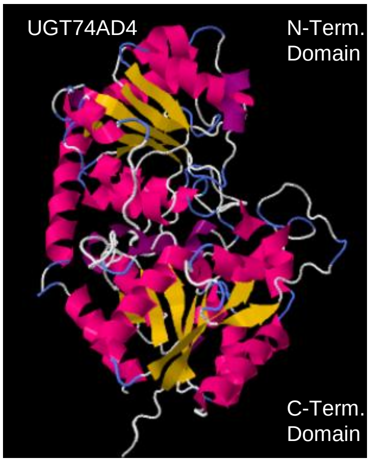

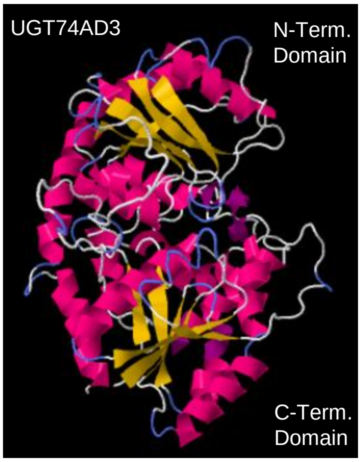

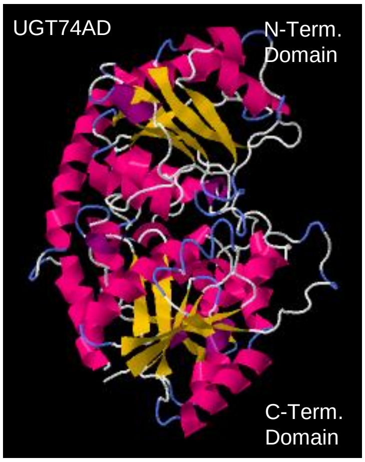

Percent Identity Matrix - created by Clustal2.1

UGT74AD2	100.00	93.89	74.61	77.11
UGT74AD3	93.89	100.00	68.37	71.33
UGT74AD4	74.61	68.37	100.00	82.22
UGT74AD5	77.11	71.33	82.22	100.00

Supplemental Figure S10. A) Multiple sequence alignment of UGT74AD amino acid sequences. B) Tridimensional models for UGT74AD enzymes and percent identity matrix
